# Development and characterization of reverse genetics systems of feline infectious peritonitis virus for antiviral research

**DOI:** 10.1186/s13567-024-01373-z

**Published:** 2024-09-27

**Authors:** Guoqian Gu, To Sing Fung, Wong Tsz Hung, Nikolaus Osterrieder, Yun Young Go

**Affiliations:** 1 Department of Infectious Diseases and Public Health, Jockey Club College of Veterinary Medicine and Life Sciences, City University of Hong Kong, Hong Kong, SAR China; 2 Department of Biomedical Sciences, Jockey Club College of Veterinary Medicine and Life Sciences, City University of Hong Kong, Hong Kong, SAR China; 3 Institut Für Virologie, Freie Universität Berlin, 14163 Berlin, Germany; 4 College of Veterinary Medicine, Konkuk University, Seoul, Republic of Korea

**Keywords:** Feline infectious peritonitis virus, reverse genetics, recombinant viruses, replicon, high-content screening, antiviral compounds

## Abstract

Feline infectious peritonitis (FIP) is a lethal, immune-mediated disease in cats caused by feline infectious peritonitis virus (FIPV), a biotype of feline coronavirus (FCoV). In contrast to feline enteric coronavirus (FECV), which exclusively infects enterocytes and causes diarrhea, FIPV specifically targets macrophages, resulting in the development of FIP. The transmission and infection mechanisms of this complex, invariably fatal disease remain unclear, with no effective vaccines or approved drugs for its prevention or control. In this study, a full-length infectious cDNA clone of the wild-type FIPV WSU79-1146 strain was constructed to generate recombinant FIPV (rFIPV-WT), which exhibited similar growth kinetics and produced infectious virus titres comparable to those of the parental wild-type virus. In addition, the superfold green fluorescent protein (*msfGFP*) and *Renilla* luciferase (*Rluc*) reporter genes were incorporated into the rFIPV-WT cDNA construct to generate reporter rFIPV-msfGFP and rFIPV-Rluc viruses. While the growth characteristics of the rFIPV-msfGFP virus were similar to those of its parental rFIPV-WT, the rFIPV-Rluc virus replicated more slowly, resulting in the formation of smaller plaques than did the rFIPV-WT and rFIPV-msfGFP viruses. In addition, by replacing the S, E, M, and ORF3abc genes with *msfGFP* and *Rluc* genes, the replicon systems repFIPV-msfGFP and repFIPV-Rluc were generated on the basis of the cDNA construct of rFIPV-WT. Last, the use of reporter recombinant viruses and replicons in antiviral screening assays demonstrated their high sensitivity for quantifying the antiviral effectiveness of the tested compounds. This integrated system promises to significantly streamline the investigation of virus replication within host cells, enabling efficient screening for anti-FIPV compounds and evaluating emerging drug-resistant mutations within the FIPV genome.

**Supplementary Information:**

The online version contains supplementary material available at 10.1186/s13567-024-01373-z.

## Introduction

Feline coronaviruses (FCoVs) belong to the genus *Alphacoronavirus* in the *Coronaviridae* family [1]. FCoVs are classified into two biotypes: feline enteric coronavirus (FECV) and feline infectious peritonitis virus (FIPV). FECV is widespread in domestic and wild cat populations and causes asymptomatic infections or enteritis with few clinical consequences. In contrast, FIPV infection causes feline infectious peritonitis (FIP), a systemic immunopathogenic disease with high mortality once clinical signs appear [2]. FECV and FIPV can be further divided into serotypes I and II. Serotype I FCoVs predominate in natural infections, accounting for 70% to 98% of cases worldwide [3–5]. Serotype II FCoVs originated from a recombination event between FCoV type I and canine coronavirus (CCoV) [6]. Unlike serotype I FCoVs, serotype II FCoVs easily grow in cell culture, making them frequently used for in vitro studies.

The FCoV genome contains 11 open reading frames (ORFs), including two large ORFs (ORF1a and ORF1b) encoding the non-structural proteins (Nsps), four structural proteins (S, E, M, N), and five accessory proteins, ORF3a, ORF3b, ORF3c, ORF7a, and ORF7b [7]. The five accessory proteins are considered important for viral replication and virulence in vivo, whereas deletion of the entire ORF3 and ORF7 genes does not affect in vitro viral replication [8]. Extensive molecular analyses have suggested that specific mutations in the S protein, ORF3c accessory protein, and possibly the ORF7b accessory protein genes of FECV alter viral tropism from enterocytes to monocytes and macrophages, playing crucial roles in the pathogenesis of systemic FIP [9–14]. Notably, the M1058L and S1060A mutations of the FCoV S protein are associated with biotype shifting, enhancing its ability to infect and adapt to monocytes and macrophages [11, 15–17]. In addition, the ORF3c protein has received considerable attention because of the prevalent deletions, frameshifts, and nonsynonymous mutations observed in the genomes of FIPV isolates. These mutations have been hypothesized to potentially enhance viral fitness and replication efficiency within the macrophages of infected animals [18]. However, notably, not all mutations within ORF3c necessarily correlate with the development of FIP. Some published studies have suggested that while these mutations may impact viral fitness, they may not directly contribute to the pathogenesis of the disease [13, 19–21]. Similarly, the ORF7b gene was initially identified as a marker of FIPV; however, it is currently deemed ineffective as an indicator of biotype switching between FIPV and FECV [22, 23]. None of the hypothesized genetic alterations associated with cellular tropism and biotype switching in FIP pathogenesis have been substantiated through in vivo experimental investigations.

Reverse genetics in virology involves manipulating and generating viral genomes from cloned DNA or RNA molecules, enabling researchers to study fundamental viral processes, develop vaccine candidates, and test antiviral drugs. Reverse genetics systems have been established for FCoV serotypes I and II, primarily aimed at generating high-titreed recombinant FCoV field isolates to investigate potential mutations associated with the biotype switch [8, 18, 24–28]. The first reverse genetics system developed for serotype II FCoV was based on targeted RNA recombination, which allows mutagenesis of only one-third of the 3ʹ end of the genome [27]. Later, the first reverse genetics system for serotype I FIPV was established by cloning the full-length cDNA into a vaccinia virus vector [29]. Infectious cDNA clones facilitate the integration of reporter genes (e.g., green fluorescence protein or luciferase) into the viral genome, generating reporter-expressing viruses. These recombinant viruses help monitor viral replication in vitro and in vivo, eliminating the need for complex titration assays. Thus, they frequently serve as effective tools for conducting high-throughput or high-content screening (HTS/HCS) of libraries, facilitating the discovery of novel antiviral compounds. Furthermore, with the evolution of reverse genetics techniques, self-replicating viral RNAs, known as replicons, were developed that retain the non-structural proteins and genetic elements necessary for producing full-length and subgenomic (sg) RNAs without producing infectious viruses. By deleting structural genes, replicons are incapable of producing infectious viral particles, allowing their manipulation within biosafety level 2 (BSL-2) facilities. This capability is important, especially when highly pathogenic viruses such as SARS-CoV, MERS-CoV, and SARS-CoV-2 are being studied [30]. Consequently, several highly pathogenic coronavirus replicons were constructed using full-length infectious cDNA clones to study viral replication and transcription in BSL-2 laboratories [31–33]. Moreover, stable cell lines expressing non-cytopathic selectable reporter replicons can be generated by inserting fluorescent or luciferase reporters with antibiotic resistance genes [34]. Hence, replicon systems offer valuable alternatives to full-length infectious cDNA clones for studying viral genome replication and conducting high-throughput screening for antiviral discovery, ensuring safety throughout the process.

In this study, we constructed a full-length infectious cDNA of FIPV WSU79-1146 (serotype II) using an in vitro ligation system that was further used to generate a recombinant rFIPV-WT virus, as well as rFIPV-msfGFP and rFIPV-Rluc viruses expressing superfold green fluorescent protein (msfGFP) and *Renilla* luciferase (Rluc) protein, respectively. Moreover, recombinant rFIPV-WT was used as the basis for constructing FIPV replicon systems. Two replicons, repFIPV-msfGFP and repFIPV-Rluc, were generated by replacing the S, E, M, and ORF3abc genes with reporter genes. Our reverse genetics systems were further utilized to assess antiviral compounds, revealing their suitability as screening platforms for evaluating such compounds. The establishment of the FIPV reverse genetics system in this study underscores its pivotal role in advancing our understanding of viral replication and pathogenesis and facilitating the development of antiviral drugs.

## Materials and methods

### Cells and chemicals

Crandell-Rees feline kidney (CRFK) cells (ATCC CCL-94, American Type Culture Collection, USA) were cultured in Dulbecco’s modified Eagle medium (DMEM, Gibco, USA) supplemented with 10% fetal bovine serum (FBS, Gibco, USA), 10 mM HEPES (Gibco, USA), and 1% penicillin–streptomycin (Gibco, USA) at 37 °C in 5% CO_2_. GS-441524, GC-376, and E64d were purchased from MCE (China). All the compounds were dissolved in DMSO as stock solutions (50 mM) and stored at −20 °C.

### Construction of infectious cDNA clones of rFIPV-WT, rFIPV-msfGFP, and rFIPV-Rluc

Viral RNA was extracted from tissue culture cells infected with FIPV WSU79-1146 virus (ATCC, VR-990) using TRIzol reagent (Thermo Fisher Scientific, USA). The extracted RNA was used for reverse transcription (RT) using the ProtoScript II First Strand cDNA Synthesis Kit (New England Biolabs, UK) following the manufacturer’s instructions. The cDNA product was stored at −20 °C until further use. The FIPV genome was amplified as seven fragments (A to G) flanked by the recognition sequence of the type IIS restriction enzyme *BsaI*. The T7 promoter was added to the 5′-end of the first nucleotide in fragment A, and a polyadenylate (A30) sequence was added to the 3′-end of the last nucleotide in fragment G. To amplify each fragment, cDNA was used as a template, and high-fidelity PCR was performed using Phusion Plus DNA Polymerase (Thermo Fisher Scientific, USA) according to the manufacturer’s instructions. The FIPVWSU79-1146 genome contained seven endogenous *BsaI* restriction sites at positions 1096, 7894, 15,222, 16,296, 24,016, 25,037 and 27,175. Among these, four *BsaI* sites were removed by introducing silent mutations at nucleotide positions C1096T, C16296G, C25037G, and C27175T by overlapping extension PCR to prevent interference with the assembly of the full-length infectious clone (Table 1). The primers used for fragment amplification and site-directed mutagenesis are summarized in Additional file 1. The PCR products were extracted using QIAquick Gel Extraction Kit (QIAGEN, Germany) and cloned and inserted into the pCR-XL-2 TOPO vector using Invitrogen TOPO XL-2 Complete PCR Cloning Kit (Thermo Fisher Scientific, USA). The resulting plasmids were pFIPV-A, -B, -C, -D, -E, -F, and -G, respectively. The monomeric superfold green fluorescent protein (*msfGFP*) coding sequence was PCR amplified using the plasmid pC035—dLwCas13a-msfGFP (Addgene 91925, Addgene, USA) as a template. To eliminate the internal *BsaI* site, two partial sequences of *Renilla* luciferase (*Rluc*) were amplified as Rluc-N and Rluc-C using the plasmid pZIKV-Rep-WT (PMID: 27658737) as a template, which was kindly provided by Prof. Pei-Yong Shi (University of Texas Medical Branch, USA). The purified hRluc-N and hRluc-C PCR products were used as templates for overlap extension PCR to generate the full-length *Rluc* gene. The partial G1 and G2 fragments were amplified from the pFIPV-G plasmid and, together with *msfGFP* or *Rluc*, were used for overlap extension PCR to produce the G-msfGFP or G-Rluc fragments. The G-msfGFP and G-Rluc fragments were subsequently cloned and inserted into the pCR-XL-2 TOPO vector (Thermo Fisher Scientific, USA) to generate the pFIPV-G-msfGFP and pFIPV-G-Rluc plasmids, respectively. The primers used for fragment amplification are summarized in Additional file 1. The constructed plasmid sequences were validated by Sanger sequencing using M13-F/R and internal primers. The plasmids were digested overnight with *BsaI* at 37 °C to recover each fragment, which was subsequently purified using QIAquick Gel Extraction Kit (QIAGEN, Germany).

**Table 1:** **Comparison of sequences between the FIPV WSU79-1146 strain and the cloned fragment**

Position (nt)	Gene	FIPV WSU79-1146 (ATCC VR-990)		Recombinant FIPV (rFIPV-WT)	
		nt	aa	nt	aa
367	ORF1a/1ab (Nsp1)	T	Proline	C	Proline
1096	ORF1a/1ab (Nsp2)	C	Leucine	T	Leucine
16 296	ORF1ab (Nsp13)	C	Valine	G	Valine
25 037	3a 3b	C C	Leucine Serine	G G	Leucine Cysteine
26 165	E	T	Isoleucine	C^a^	Threonine
27 175	N	C	Aspartate	T	Aspartate

^a^Same as the NCBI reference sequence (NC_002306) but different from the sequence of ATCC VR-990.

To assemble the full-length infectious cDNA, fragments A–D and E–G (G-msfGFP for rFIPV-msfGFP and G-Rluc for rFIPV-Rluc) were ligated using T4 ligase (New England Biolabs, UK) in separate reactions and incubated at 4 °C overnight. The resulting mixtures were subsequently combined and further incubated at 4 °C overnight. The full-length infectious cDNA of the recombinant FIPV (rFIPV-WT, rFIPV-msfGFP, and rFIPV-Rluc) was then extracted using phenol chloroform. In addition, the FIPV N gene with a 5′-T7 promoter and a 3′-polyadenylate (A30) was produced by Phusion PCR using pFIPV-G as a template. The N gene PCR amplicon was purified with a MiniBEST DNA Fragment Purification Kit (TaKaRa, Japan).

### Construction of FIPV replicons, repFIPV-msfGFP, and repFIPV-Rluc

To produce the replicon systems, two plasmids were generated by replacing the S, E, M, and ORF3abc genes with the reporter *msfGFP* and *Rluc* genes. Briefly, the pFIPV-F and pFIPV-G plasmids were used as templates for amplifying partial F3 and G4 fragments, respectively. The sequence of the foot-and-mouth disease virus 2A peptide (F2A) fused to the neomycin resistance gene (NeoR) was amplified using pcDNA3.1( +) as a template in two consecutive rounds of PCR to produce the fusion fragment F2A-NeoR. The *msfGFP* and *Rluc* genes were amplified along with F2A-NeoR by overlap extension PCR to generate the fusion fragments msfGFP-F2A-NeoR and Rluc-F2A-NeoR, respectively. The purified PCR products of the partial F3 fragment, msfGFP-F2A-NeoR, and partial G4 were used as templates for overlap extension PCR and were subsequently cloned and inserted into the pCR-XL-2 TOPO vector (Thermo Fisher Scientific, USA). The resulting plasmids were named pFIPV-R-msfGFP and pFIPV-R-Rluc. To recover the R-msfGFP and R-Rluc fragments, pFIPV-R-msfGFP and pFIPV-R-Rluc were digested overnight with *BsaI* at 37 °C, and the digested products were purified using QIAquick Gel Extraction Kit (QIAGEN, Germany) following agarose gel electrophoresis.

Fragments A–C and fragments D, E R-msfGFP or R-Rluc were ligated in a separate reaction. After incubation at 4 °C overnight, the two ligation reactions were combined and further incubated at 4 °C overnight. The full-length repFIPV-msfGFP and repFIPV-Rluc DNAs were then extracted using phenol chloroform.

### In vitro transcription of RNA and electroporation

The full-length cDNA and replicon DNA constructs were used to generate in vitro transcribed (IVT) RNAs using the mMESSAGE mMACHINE T7 Transcription Kit (Thermo Fisher Scientific, USA) according to the manufacturer's instructions. Briefly, a 50 μL reaction was set up by adding 1 μg of DNA template and 7.5 μL of GTP (cap analogue-to-GTP ratio of 1:1) and incubating at 32 °C for 5 h. IVT RNA was obtained by phenol chloroform extraction followed by isopropanol precipitation. The FIPV N gene transcript was transcribed in vitro the mMESSAGE mMACHINE T7 Transcription Kit with a 2:1 ratio of cap analogue to GTP and electroporated together with the full-length RNA and replicon RNA to increase the replication of the recombinant viruses and replicon [35]. The resulting RNA transcripts, consisting of 10 μg of total RNA mixed with 20 μg of N gene transcripts, were electroporated into 2 × 10^6^ CRFK cells resuspended in 0.2 mL of Ingenio® Electroporation Solution in a 4-mm cuvette, using Gene Pulser X-cell system (Bio-Rad, USA) at 450 V, 50 μF, and infinite resistance, following previously described methods with modifications [36]. After 5 min of recovery at room temperature, the cells were seeded in a 6-well plate and incubated at 37 °C with 5% CO_2_. The cell culture supernatant was harvested at 48 to 72 h post-electroporation when extensive cytopathic effects (CPEs), such as multinucleation and cell sloughing, were observed. The identification of the recombinant viruses was validated by sequencing the genetic markers shown in Additional file 1.

### Confirmation of the full-genome sequences of the recovered recombinant viruses using next-generation sequencing (NGS)

The genomes of the recovered recombinant viruses (passage 0) were fully sequenced using next-generation sequencing (NGS). Viral RNA was extracted from cell culture supernatants using the TRIzol reagent (Thermo Fisher Scientific, USA). First and second-strand cDNAs were then synthesized using the NEBNext Ultra RNA Prep Kit (New England Biolabs, UK) with random primers, following the manufacturer’s instructions. cDNA libraries were prepared using the Nextera DNA Sample Preparation Kit (Illumina, CA, USA) for sequencing on an Illumina platform. After sequencing, the data were processed by demultiplexing, trimming adapters, and removing low-quality reads. The processed reads were aligned to reference sequences using the BWA software [37]. The average sequencing depth was calculated by dividing the genome into 100 bp bins and applying the Giraffe pipeline for analysis [38]. Variants were identified relative to the reference sequence using LoFreq with default settings [39].

### Virus propagation

For the preparation of virus stocks, CRFK cells were infected with FIPV WSU79-1146 (ATCC, VR-990), rFIPV-WT, rFIPV-msfGFP or rFIPV-Rluc viruses at MOI 0.1, and cultured in 2% FBS containing DMEM at 37 °C with 5% CO_2_ for 48 h. After three freeze/thaw cycles, cell lysates were clarified by centrifugation at 1500 × *g* at 4 °C for 30 min. The virus stocks were titrated by plaque assay, aliquoted, and stored at −80 °C until further use. Passage 1 (P1) virus stocks of recombinant viruses (rFIPV-WT, rFIPV-msfGFP, rFIPV-Rluc) and FIPV WSU79-1146 P3 were used for subsequent experiments.

### Plaque assay

Confluent monolayers of CRFK cells seeded on 6-well plates were infected with tenfold serially diluted wild-type FIPV or recombinant viruses (rFIPV-WT, rFIPV-msfGFP, and rFIPV-Rluc). The plates were agitated every 10–15 min to ensure proper coverage. After 1 h of adsorption, unbound viruses were removed by washing the cells twice with ice-cold PBS. Subsequently, overlay medium (0.4% agarose in DMEM) was added to each well and incubated at 37 °C with 5% CO_2_. After 72 h of incubation, cells were fixed with 4% formaldehyde and stained with crystal violet. The plates were then scanned as 8-bit grayscale images using ChemiDoc MP Imaging System (Bio-Rad, USA), and the areas of plaques were determined using the ImageJ software (National Institutes of Health, USA).

### Luciferase assay

The *Renilla* luciferase activity was measured by the *Renilla* luciferase assay system (Promega, USA) using SpectraMax iD3 Multi-Mode Microplate Readers (Molecular Devices, USA). In brief, 100 µL of 1 × *Renilla* luciferase substrate was mixed with 20 µL of cell lysate in an F96 MicroWell black plate (Thermo Fisher Scientific, USA), and luciferase signals were collected for ten seconds after a two-second incubation.

### RNA extraction, reverse transcription-PCR (RT-PCR), and Sanger sequencing

To evaluate the genetic and phenotypic stability of the recombinant viruses with reporter genes, the rFIPV-msfGFP and rFIPV-Rluc were passaged in CRFK cells up to ten passages. CRFK cells seeded in 12-well plates were infected with rFIPV-msfGFP or rFIPV-Rluc at MOI 0.1. Unbound viruses were removed after 2 h, and the cells were replaced with plain DMEM following two rounds of washing with PBS. When complete CPE was observed, the supernatant was collected and clarified by centrifugation at 1500 × *g* at 4 °C for 10 min. The harvested viruses were used for the next round of passages after titration by plaque assay.

For sequencing of *msfGFP* and *Rluc* genes, total RNA was extracted using TRIzol reagent (Thermo Fisher Scientific, USA). The extracted RNA was used for reverse transcription (RT) using the ProtoScript II First Strand cDNA Synthesis Kit (New England Biolabs, UK) following the manufacturer's instructions. To amplify the *msfGFP* and *Rluc* flanking regions, the cDNA products served as templates, and high-fidelity PCR was performed using Phusion Plus DNA Polymerase (Thermo Fisher Scientific, USA) with specific primers for amplification of *msfGFP* and *Rluc* genes (Additional file 1). The PCR products were purified by MiniBEST DNA Fragment Purification Kit (TaKaRa, Japan) and sent for Sanger sequencing. The sequencing results were aligned with the expected sequences using the Snapgene software (USA).

### Cell viability

CRFK cells were seeded in 96-well plates at a density of approximately 2 × 10^4^ cells/well. After overnight incubation, serially diluted compounds or DMSO (vehicle) ranging from 100 μM to 0.001 μM were added to each well and incubated at 37 ℃ in a 5% CO_2_ incubator. Cell viability was measured after 48 or 72 h incubation using the CellTiter 96 AQueous One Solution cell proliferation assay (MTS; Promega, USA), according to the manufacturer’s instructions. Absorbance at 490 nm was measured using SpectraMax iD3 Multi-Mode Microplate Readers (Molecular Devices, USA).

### Antiviral assay

CRFK cells were seeded in 96-well plates at a density of approximately 2 × 10^4^ cells/well. After overnight incubation, CRFK cells were infected by wild-type FIPV, rFIPV-msfGFP, or rFIPV-Rluc viruses. Subsequently, threefold serially diluted compounds (GS-441524 and GC-376) or DMSO were added and incubated at 37 ℃ in a 5% CO_2_ incubator. For the rFIPV-msfGFP-based antiviral assay, the number of GFP-positive cells was quantified using high-content imaging systems. The inhibition rate was calculated using the formula below, where *n* represents the number of GFP-positive cells in compound-treated samples, and *N* represents the number of GFP-positive cells in DMSO-treated samples.

$$i=\left(1- \frac{n}{N }\right)\times 100\%$$

High-content image acquisition and analyses were performed using the high-content module NIS-Elements AR (Version 5.42.02. Nikon) of the Ti2 fluorescence microscope (Nikon, Japan). A 4 × objective was used with the GFP channel and the DAPI channel. An in-house algorithm was created for cell counting, segmentation, and validation to calculate the percentage of infected cells. For the antiviral assay using the rFIPV-Rluc virus, the *Rluc* activity was measured. The inhibition rate was determined relative to the control cells treated with DMSO. The inhibition rate was calculated using the formula below, where *r* represents the luciferase readings from compound-treated cells, and *R* represents the luciferase readings from DMSO-treated cells.

$$i=\left(1- \frac{r}{R }\right)\times 100\%$$

For the replicon-based assay, CRFK was transfected with repFIPV-msfGFP or repFIPV-Rluc RNA, as described above. Subsequently, the threefold serially diluted compounds (GS-441524, GC-376, and E64d) were added and incubated at 37 ℃ in a 5% CO_2_ incubator. After 36 h post-transfection, for the repFIPV-msfGFP-based assay, the amount of the *msfGFP* expressing cells was measured by high-content imaging systems, and the inhibition rate was calculated relative to that of DMSO-treated cells. For the repFIPV-Rluc-based assay, the *Rluc* activity of the transfected cells was measured, and the inhibition rate was calculated relative to that of DMSO-treated cells after 36 h post-transfection. Cell viability was measured after 48 or 72 h incubation using the CellTiter 96 AQueous One Solution cell proliferation assay (MTS; Promega, USA), according to the manufacturer’s instructions. Absorbance at 490 nm was measured using SpectraMax iD3 Multi-Mode Microplate Readers (Molecular Devices, USA). The EC_50_ and CC_50_ values were calculated using the nonlinear regression curve fit in GraphPad Prism software (USA). The plotted data represented the means and standard deviations (s.d.) from three independent experiments.

### Statistical analysis

The one-way ANOVA method was used to analyse the significant difference between the indicated samples and the respective control samples. Significance levels were presented by the *p* value (ns, non-significant; ^*^p < 0.05; ^**^p < 0.01; ^****^p < 0.0001).

## Results

### Characterization of rFIPV viruses expressing *msfGFP* and *Rluc* genes

**Figure 1:**
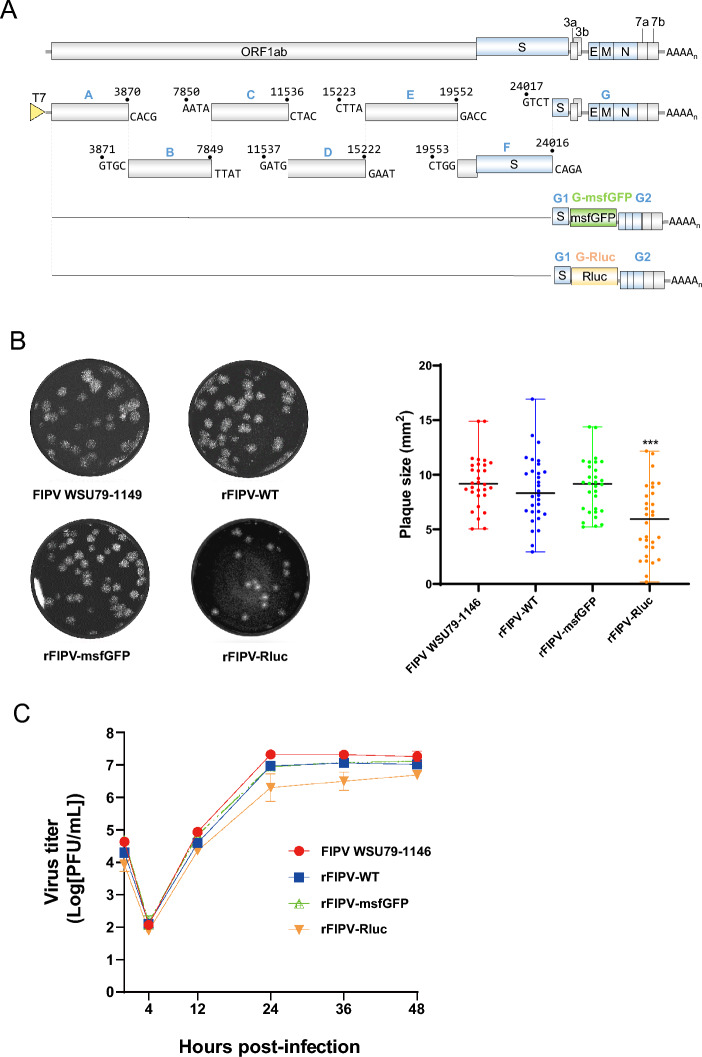
**Construction and characterization of the rFIPV-msfGFP and rFIPV-Rluc viruses**. **A** Schematic diagram showing the genomic structures of the rFIPV-WT, rFIPV-msfGFP, and rFIPV-Rluc viruses used in this study and the strategy for constructing the recombinant viruses. The ORF3abc genes in the FIPV genome were replaced with *msfGFP* and *Rluc* genes. The full-length FIPV genome was divided into seven fragments. The seven fragments and the T7 promoter were subsequently cloned and inserted into the PCR-XL-2 vector by the TOPO cloning assay, as a *BsaI* restriction site flanked each fragment. The PCR-XL-2 carrying each fragment was digested with *BsaI* and ligated with the T4 ligation enzyme. The full-length genomic cDNA and N gene cDNA were transcribed into RNA in vitro and transfected into CRFK cells via electroporation. The schematic diagram is not drawn to scale. **B** Morphology and size of viral plaques of the rFIPV-WT, rFIPV-msfGFP, and rFIPV-Rluc strains compared with those of the parental FIPV WSU79-1146 strain. Representative images of the plaque morphology of the above three recombinant viruses and the wild-type FIPV WSU79-1146 are shown. The areas of 30 plaques were determined and plotted for each virus. The experiment was repeated three times with similar results, and the results of one representative experiment are shown. ***p < 0.001. **C** Growth kinetics of the recombinant viruses. CRFK cells were infected with recombinant viruses and wild-type FIPV WSU79-1146 at an MOI of 0.1. Viral titres from culture supernatants at the indicated time points were determined by plaque assays. The error bars indicate the means and standard deviations from three independent experiments.

In this study, a full-length infectious cDNA clone of the wild-type FIPV WSU79-1146, namely, rFIPV-WT, was generated using an in vitro ligation approach. Briefly, the full-length RNA genome of the wild-type FIPV WSU79-1146 strain was amplified via RT‒PCR into seven cDNA fragments and subsequently cloned and inserted into the pCR-XL-2 vector using TOPO cloning with *BsaI* restriction sites for directional assembly. After digestion, the seven fragments were ligated and assembled into a full-length infectious cDNA clone of FIPV WSU79-1146 (Additional files 2A-C). Four synonymous nucleotide mutations at endogenous *BsaI* sites were introduced to serve as markers to distinguish the infectious clone-derived virus from the parental isolate (Table 1). To establish the reporter infectious cDNA clones of FIPV WSU79-1146, the ORF3abc coding sequences were replaced with *msfGFP* or *Rluc* genes, resulting in the generation of rFIPV-msfGFP and rFIPV-Rluc genome-length RNA (Additional files 2D-G and Figure 1A). To recover the rFIPV-WT, rFIPV-msfGFP, and rFIPV-Rluc viruses, the genome-length in vitro transcribed RNAs were electroporated into CRFK cells. The rFIPV-WT, rFIPV-msfGFP, and rFIPV-Rluc viruses were successfully recovered. Viral RNA isolated from the recovered viruses was sequenced via next-generation sequencing (NGS). The sequencing results confirmed the presence of the expected synonymous mutations at positions 1096, 16,296, 25,037, and 27,175 in the rFIPV-WT genome, verifying that the obtained sequence was derived from rFIPV-WT (Table 1). The correct insertion of the reporter genes (*msfGFP* and *Rluc*) at the original ORF3abc locus was also confirmed (Additional file 3). Most variants identified in the three recombinant viruses were found at relatively low frequencies (< 5%) throughout the genome, indicating stable integration of the reporter genes and minimal genomic alterations between the recovered recombinant virus sequences and the electroporated full-length RNA sequences (Additional file 3). Next, the replication properties of the recombinant and wild-type parental viruses were compared. The plaque sizes of the rFIPV-WT and rFIPV-msfGFP viruses were similar to that of the wild-type parental FIPV, whereas the average plaque size of the rFIPV-Rluc virus was significantly smaller (Figure 1B). The one-step growth curve of the recombinant viruses was further analysed for growth kinetics. The replication kinetics of rFIPV-WT were similar to those of its parental FIPV, with peak titres of ~ 10^7^ PFU/mL at 24 hpi (Figure 1C), suggesting that the silent mutations at endogenous *BsaI* sites did not affect viral replication. To assess the impact of the reporter genes, the replication properties of the reporter viruses were compared with those of the wild-type parental FIPV in CRFK cells. Comparable replication kinetics were noted for rFIPV-msfGFP, whereas the replication of the rFIPV-Rluc virus was slower, with a titre approximately 1 log lower than that of the wild-type parental FIPV at 24 hpi. By 48 hpi, the rFIPV-Rluc virus reached a virus titre comparable to that of both the rFIPV-WT and the rFIPV-msfGFP viruses. Both the rFIPV-WT and the rFIPV-msfGFP viruses demonstrated replication kinetics akin to those of the wild-type parental FIPV WSU79-1146 strain. In contrast, the rFIPV-Rluc virus replicated more slowly and produced smaller plaques. These findings indicate a potential adverse effect of *Rluc* gene insertion on an early stage of rFIPV-Rluc viral replication. Nevertheless, it was determined that ORF3abc is not essential for in vitro genome replication.

### Genetic stability of the rFIPV-msfGFP and rFIPV-Rluc viruses

**Figure 2:**
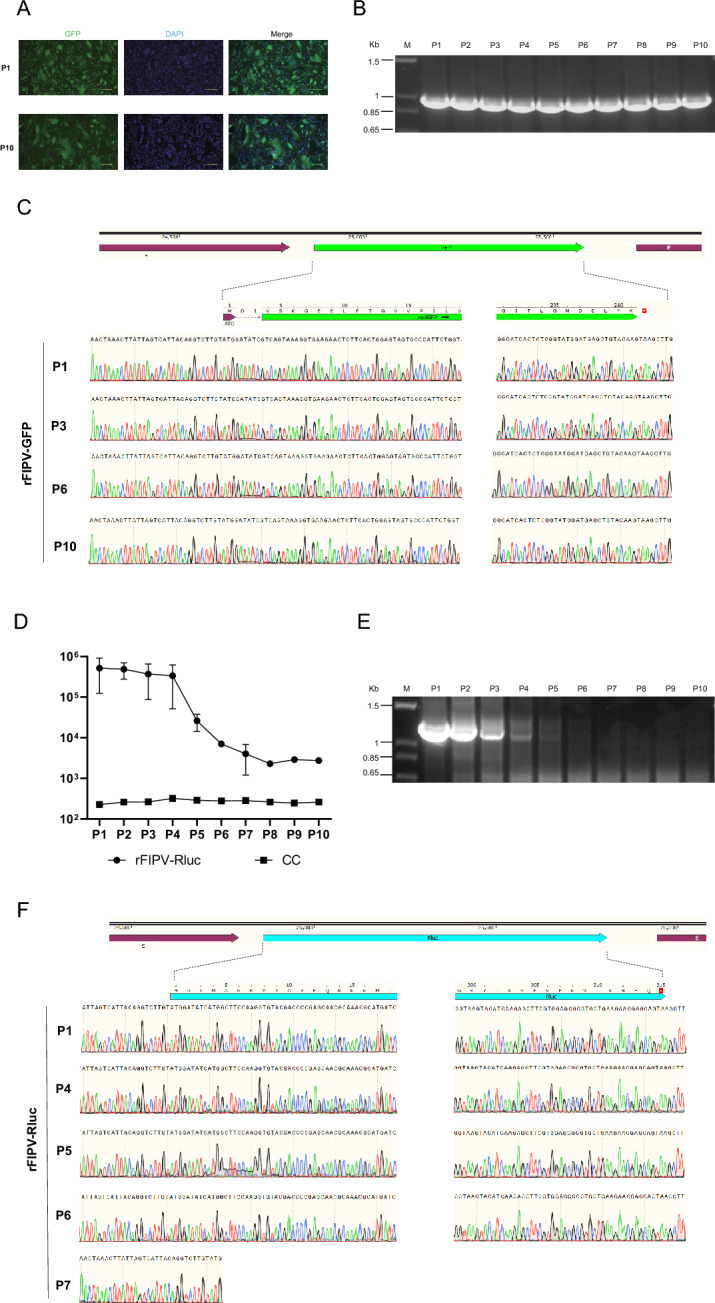
**Genetic stability of the rFIPV-msfGFP and rFIPV-Rluc viruses.** The rFIPV-msfGFP and rFIPV-Rluc viruses were serially passaged at an MOI of 0.1 in CRFK cells ten times. **A** Representative fluorescence images of rFIPV-msfGFP virus-infected CRFK cells during passage. P1 and P10 are shown. **B** Analysis of the genetic stability of the rFIPV-msfGFP virus after ten passages. Viral RNA was extracted from the culture supernatants of each passage, and RT-PCR was performed with a primer set flanking the *msfGFP* gene. The resulting RT-PCR products were resolved by 1% agarose gel electrophoresis. The 1-kb DNA ladders are indicated. **C** Chromatograms of Sanger sequencing results of the *msfGFP* gene of the rFIPV-msfGFP virus from P1, 3, 6, and 10 are shown. **D** Rluc activity of rFIPV-Rluc at each passage. For each passage, the cell lysate samples were subjected to luminescence analysis. Uninfected cells served as the cell control (CC). The error bars indicate the means and standard deviations from three independent experiments. **E** Analysis of the genetic stability of the rFIPV-Rluc virus after ten passages. Viral RNA was extracted from culture supernatants of each passage, and RT-PCR was performed with a primer set flanking the *Rluc* gene. The resulting RT-PCR products were resolved by 1% agarose gel electrophoresis. The 1-kb DNA ladders are indicated. **F** Chromatograms of Sanger sequencing results of the *Rluc* gene of the rFIPV-Rluc virus from P1, 4, 5, 6, and 7 are shown.

To assess the extended stability of the reporter genes in the rFIPV-msfGFP and rFIPV-Rluc viruses, the reporter viruses were passaged ten times in CRFK cells. Fluorescence imaging was conducted after each passage of the rFIPV-msfGFP virus to evaluate the expression of the GFP reporter in infected cells. The stability of the *msfGFP* gene in rFIPV-msfGFP was observed for up to ten passages, as demonstrated by comparable numbers of msfGFP-positive cells at passages 1 (P1) and P10 (Figure 2A). RT-PCR was subsequently conducted to confirm the presence of the *msfGFP* gene in the viral genome from P1 to P10, utilizing two primers targeting the flanking regions of the inserted gene. As anticipated, the RT-PCR analysis confirmed the presence of RT-PCR products of the expected size (999 bp) containing the flanking regions and the complete *msfGFP* gene from P1 to P10 of the rFIPV-msfGFP viral genomes (Figure 2B). Additionally, Sanger sequencing verified the conservation of the *msfGFP* gene and its flanking region from P1 to P10 (Figure 2C). In the case of the rFIPV-Rluc virus, the luminescence signal remained relatively stable for the initial four passages but exhibited a sharp decrease of 1.5 log at P5 and remained low in subsequent generations (Figure 2D). Furthermore, the band of the *Rluc* gene started to decline from P2 and was absent by P7 (Figure 2E). Sanger sequencing indicated that the *Rluc* gene remained intact from P1 to P6. However, by P7, only the start codon of the *Rluc* gene was retained, with the remainder of the gene deleted (Figure 2F). In summary, the results suggest that rFIPV-msfGFP remained stable throughout ten passages in cell culture, whereas the stability of rFIPV-Rluc was limited to only four passages.

### Development of antiviral assays utilizing reporter rFIPV-msfGFP and rFIPV-Rluc viruses

**Figure 3:**
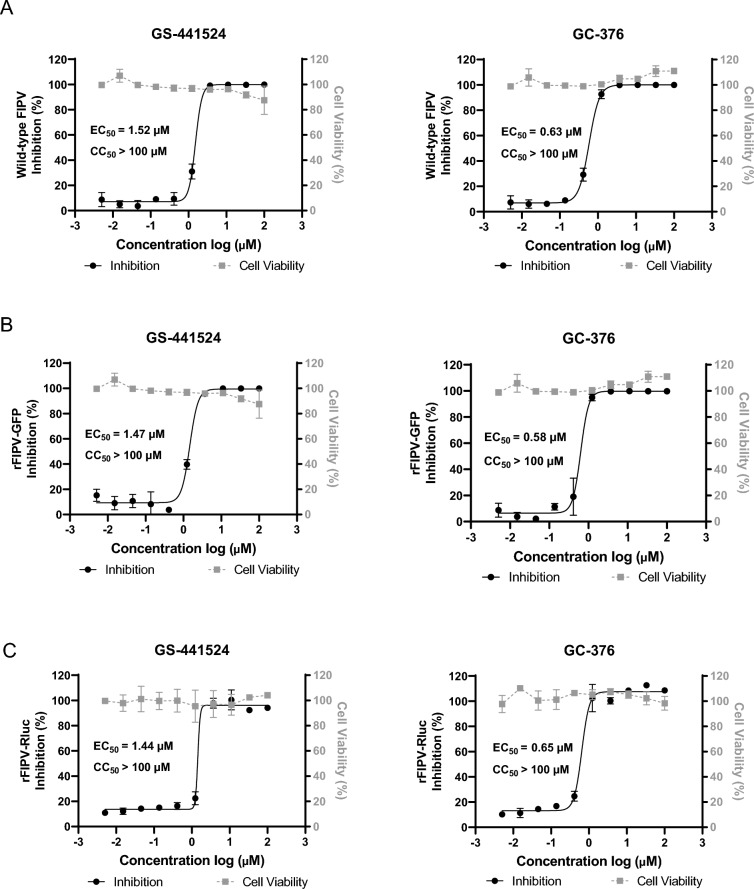
**Application of the rFIPV-msfGFP and rFIPV-Rluc viruses in drug screening.** **A** Antiviral assay of GS-441524 or GC-376 using wild-type FIPV and their cytotoxic effects. Antiviral assay of GS-441524 or GC-376 using **B** rFIPV-msfGFP and **C** rFIPV-Rluc. Relative inhibition was calculated on the basis of reporter gene expression in cells treated with antiviral drugs compared with that in cells treated with DMSO. EC_50_ values and CC_50_ values were calculated as explained in the Materials and Methods and are represented on individual graphs. The error bars indicate the means and standard deviations from three independent experiments.

Next, the efficacy of the rFIPV-msfGFP and rFIPV-Rluc viruses in antiviral screening assays was assessed using previously reported FIPV inhibitors, GS-441524 (a polymerase inhibitor) and GC-376 (an inhibitor of 3CLpro), as reference compounds and compared with that of the wild-type FIPV WSU79-1146. The infection parameters, including the readout time (48 h and 72 h) and MOIs (0.01 and 0.001), were evaluated for the two reporter viruses. Z’ factor analysis revealed that an MOI of 0.01 and 48 h of incubation were optimal for the subsequent antiviral experiments, as evidenced by a Z’ factor exceeding 0.5 (Additional files 4 A and B). Under the optimized conditions, the results confirmed that GS-441524 and GC-376 inhibited the replication of the wild-type FIPV WSU79-1146, with EC_50_ values of 1.52 μM and 0.63 μM, respectively (Figure 3A). Similarly, the EC_50_ values for GS-441524 and GC-376 using the rFIPV-msfGFP virus were 1.47 μM and 0.58 μM, respectively (Figure 3B), whereas for the rFIPV-Rluc virus, the EC_50_ values were 1.44 μM and 0.65 μM, respectively (Figure 3C). Importantly, the EC_50_ values determined using the reporter viruses closely mirrored those obtained with the wild-type FIPV for both reference compounds. We further investigated the relationship between the inhibition rate and virus titre. As a result, treatment with GS-441524 at concentrations of 3.7 μM or higher led to a reduction in rFIPV-msfGFP virus titre of more than 4 log, with antiviral activity reaching its maximum within this concentration range. Similarly, treatment with 3.7 μM to 100 μM GC-376 achieved 100% antiviral activity and resulted in a 4–5 log reduction in rFIPV-msfGFP virus titres (Additional file 5A). Comparable results were observed with rFIPV-Rluc (Additional file 5B). These findings indicate a close correlation between antiviral activity and virus production inhibition, suggesting that assays using these recombinant reporter viruses offer a reliable and efficient alternative to traditional virus titre methods, significantly saving time and resources. These results underscore the validity of recombinant reporter viruses, particularly in facilitating high-throughput screening of antivirals. These findings collectively suggest that recombinant reporter viruses could be reliably utilized for studying FIPV replication and screening antiviral inhibitors on a larger scale.

### Construction and characterization of the FIPV replicons repFIPV-msfGFP and repFIPV-Rluc

**Figure 4:**
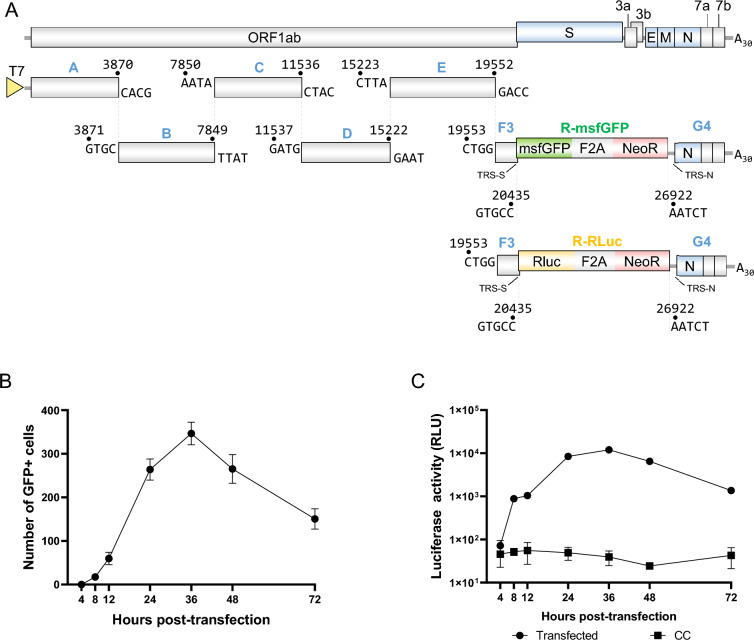
**Construction of repFIPV-msfGFP and repFIPV-Rluc.** **A** Schematic diagram showing the genomic structures of the FIPV replicons used in this study and the strategy for constructing the replicons. The FIPV replicon genomic cDNA and N gene cDNA were transcribed into RNA in vitro and transfected into CRFK cells via electroporation. **B** Kinetics of *msfGFP* gene expression postelectroporation in CRFK cells. The number of GFP-positive cells in each well containing 4 × 10^4^ cells was counted at the indicated time points. The error bars indicate the means and standard deviations from three independent experiments. **C** Kinetics of *Rluc* gene expression post-electroporation in CRFK cells. For each time point, the cell lysate samples were subjected to luminescence analysis. The untransfected cells served as the CC. The error bars indicate the means and standard deviations from three independent experiments.

Two FIPV replicons containing *msfGFP* or *Rluc* genes were developed via the same strategy as the recombinant rFIPV-WT virus (Additional file 6). In brief, the reporter *msfGFP* or *Rluc* genes were inserted downstream of FIPV ORF1b, with subgenomic mRNA expression driven by the FIPV transcription regulatory sequence (TRS) of the S gene (Figure 4A). Following in vitro transcription of the full-length repFIPV-msfGFP, RNA was electroporated into CRFK cells. The expression of the reporter genes was then monitored at various time points. As depicted in Figure 4B, msfGFP expression was observed in cells after 8 h of transfection, peaking at 36 h post-transfection (hpt) and decreasing thereafter. Similarly, transfection with repFIPV-Rluc RNA led to increased luciferase activity, with the luminescence signal reaching its peak at 36 hpt (Figure 4C). These findings confirm the expression of reporter proteins driven by the S TRS following the transfection of the replicon constructs into CRFK cells. Overall, these results demonstrate the functionality of the repFIPV-msfGFP and repFIPV-Rluc systems in replicating within cells, providing a convenient and sensitive approach for analysing viral genome replication.

### Evaluation of FIPV replicons as tools for drug discovery

**Figure 5:**
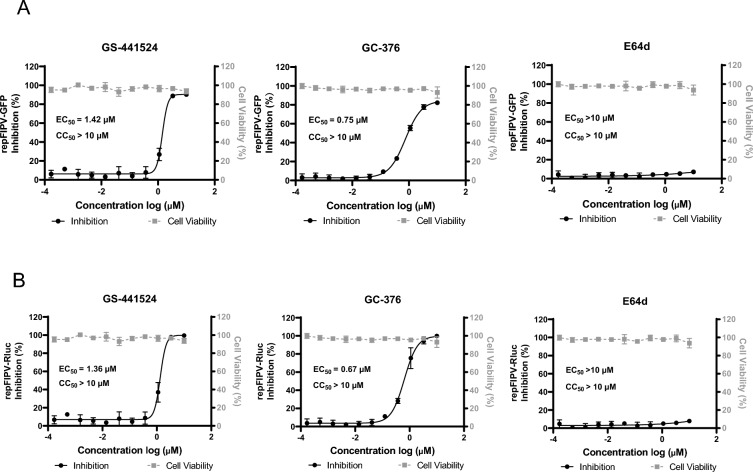
**Application of FIPV replicons in drug discovery**. Dose-dependent responses of **A** repFIPV-msfGFP and **B** repFIPV-Rluc reporter activity to GS-441524, GC-376, and E64d. Inhibition was determined by the percentage of reporter gene signals compared with that in DMSO-treated cells. The cytotoxicity of GS-441524, GC-376, and E64d to the cells was also examined. EC_50_ values and CC_50_ values were calculated as explained in the Materials and Methods and are represented on individual graphs. The error bars indicate the means and standard deviations from three independent experiments.

The suitability of repFIPV-msfGFP and repFIPV-RLuc as tools for antiviral screening was further investigated. GS-441524 and GC-376 were used as positive reference controls, whereas E64d, a nonspecific cysteine inhibitor, was used as a negative control. E64d blocks the release of viral RNA into the cell by inhibiting the breakdown of S proteins by cathepsins in the endosome/lysosome system, which is unlikely to interfere with the replication of the viral replicon. The inhibition rate was assessed by measuring the reduction in reporter gene expression compared with that in cells treated with DMSO at 36 hpt. Figure 5A shows a dose-dependent decrease in the msfGFP signal following treatment with GS-441524 and GC-376 after repFIPV-msfGFP RNA transfection. The EC_50_ values of GS-441524 and GC-376 were 1.42 μM and 0.75 μM, respectively, which were consistent with those determined by the rFIPV-msfGFP virus-based assay (Figure 3B). In contrast, E64d had no inhibitory effect on the expression of msfGFP (Figure 5A). The EC_50_ values of GS-441524 and GC-376 determined by repFIPV-Rluc were 1.36 μM and 0.67 μM (Figure 5B), respectively, which were consistent with those of the rFIPV-Rluc virus-based assay (Figure 3C). Similarly, no reduction in *Rluc* enzyme activity was observed with E64d treatment (Figure 5B). These results suggest that E64d does not interfere with viral RNA synthesis. In addition, no cytotoxic effects were observed in the treated cells at any concentration (Figures 5A and B). In summary, the results obtained using compounds with established antiviral mechanisms validate the applicability of these replicons for identifying antiviral compounds, particularly those targeting specific steps in the viral genome replication process.

## Discussion

In this study, we have established the in vitro ligation‐based reverse genetics systems for the FIPV WSU79-1146 strain to produce recombinant viruses and replicons referring to the methods used during the COVID-19 pandemic for constructing SARS-CoV-2 infectious clones and replicons [36, 40]. Currently, several reverse genetics systems for coronaviruses have been developed employing diverse methodologies, such as (i) in vitro ligation of subgenomic fragments [35], (ii) transformation-associated recombination (TAR) [41], or (iii) cloning in a bacterial artificial chromosome (BAC) or yeast artificial chromosome (YAC) [42, 43]. BAC-based strategies were utilized to successfully establish infectious clones for various coronaviruses [40], relying on traditional cloning techniques to create a one-plasmid system capable of functioning as infectious full-length viral cDNA clones or replicons. Although constructing a BAC-based system demands substantial effort, resulting clones are easily manipulable, akin to regular plasmids. In comparison to the BAC-based strategy, the in vitro ligation-based approach offers distinct advantages. It is relatively straightforward, leveraging conventional cloning techniques on high or medium-copy plasmids for easy amplification and mutagenesis. Moreover, this strategy addresses instability issues commonly associated with some coronavirus cDNA sequences by dividing toxic regions into multiple segments [44].

To construct the reporter rFIPV-msfGFP and rFIPV-Rluc viruses, the ORF3abc genes of FIPV WSU79-1146 were replaced by *msfGFP* and *Rluc* genes, respectively. Both reporter recombinant viruses were successfully rescued. Consistent with previous findings [8], the rescued rFIPV-msfGFP virus exhibited similar kinetics to its parental wild-type virus. In contrast, the rFIPV-Rluc virus displayed slower replication and formed smaller plaques than the rFIPV-WT and rFIPV-msfGFP viruses, suggesting a potential decline in viral fitness upon insertion of the *Rluc* gene. Furthermore, the inserted *Rlu*c gene was observed to be deleted by P7, while the *msfGFP* gene remained stable after ten passages. Previous studies indicated that the genetic stability of recombinant avian coronavirus infectious bronchitis virus (IBV) carrying exogenous genes was influenced by the size, intrinsic characteristics, and genomic insertion site [45]. Given that both *msfGFP* and *Rluc* genes were inserted in the same location of the genome, it can be inferred that the instability is attributed to the *Rluc* gene. The relative instability of the *Rluc* gene compared to the *msfGFP* gene could be due to its larger size, as larger genes are generally more susceptible to deletions. Additionally, foreign genes may contain sequences that render them more vulnerable to mutation or recombination during coronavirus replication, as recombination events within a foreign gene depend on the degree of base pairing between the foreign gene and the recombinant virus genome sequence [46]. For instance, the instability observed in recombinant mouse hepatitis virus (MHV) due to the presence of the firefly luciferase gene may be associated with its higher GC content (46.7%) compared to the coronavirus genome (MHV, 41.8%) [46]. Similarly, we observed that the rFIPV-Rluc virus is less stable compared to the rFIPV-msfGFP virus, which may be associated with the higher GC content (54.9%) of the *Rluc* gene compared to those of the *msfGFP* gene (45.3%) and FIPV genome (38.1%). Indeed, it has been demonstrated in poliovirus vectors that manipulation of GC contents can enhance the genetic stability of foreign inserts [47]. Therefore, the stability of the rFIPV-msfGFP virus in comparison to rFIPV-Rluc facilitates its application in long-term studies, rendering it a superior research tool.

In parallel, the transient FIPV replicon systems derived from the infectious cDNA clones were established, marking the first replicon system for serotype II FIPV. The minimum requirements for autonomous replication of coronavirus RNA include the presence of 5ʹ- and 3ʹ-UTRs, the replicase gene (ORF 1a and 1b), and the nucleocapsid gene [48]. Therefore, we designed the replicon systems of FIPV, namely, repFIPV-msfGFP and repFIPV-Rluc, by replacing the structural S, E, and M and the accessory ORF3abc genes of the infectious cDNA clone with *msfGFP* and *Rluc*. The expression of *msfGFP* and *Rluc* genes was observed at 8 hpt and peaked at 36 hpt, which indicated that both replicons replicated in CRFK cells. Furthermore, we attempted to establish stable replicon cell lines expressing repFIPV-msfGFP and repFIPV-Rluc, respectively. However, we have been unsuccessful in achieving this goal. A recent report suggested that establishing stable SARS-CoV-2 replicon cells was unsuccessful due to the toxicity derived from a viral protein or a replication step [49]. The non-structural protein 1 (Nsp1) of coronaviruses, translated from replicon sgRNAs, has been demonstrated to possess viral cytotoxic properties [50–52]. Nsp1 plays a crucial role in the shut-off of host protein synthesis during SARS-CoV-2 replication [53], and its expression has been shown to induce cell cycle arrest and cell death [53, 54]. Therefore, the cytotoxic effects induced by Nsp1 in replicon-expressing cells are believed to hinder the successful establishment of stable replicon cell lines. Recent investigations have highlighted the role of specific mutations (K164A/H165A) in Nsp1 of SARS-CoV-2 in mitigating cellular toxicity mediated by Nsp1 [49]. The K164A/H165A mutation in Nsp1 allowed for the successful establishment of a stable SARS-CoV-2 replicon cell line on BHK-21 cells after selection [34], which is consistent with the structural analysis suggesting that this mutation decreases the interaction between Nsp1 and the ribosome, leading to enhanced accessibility of the ribosome to host mRNA and mitigating Nsp1-induced toxicity to the host [55]. Shen et al. reported similar findings indicating that a specific motif (amino acids 91–95, Ala-Asn-Cys-Asn-Gly) within the transmissible gastroenteritis virus (TGEV) Nsp1 protein is a conserved region involved in inhibiting host protein synthesis [56]. Substituting the motif with a flexible Ser-Gly-Ser-Gly-Ser linker did not impair virus replication in cell culture but significantly reduced virulence in vivo. This motif was also identified within the FIPV genome [56]. Introducing similar mutations into the Nsp1 of FIPV could mitigate the cytotoxic effects induced by Nsp1 in cells transfected with replicons, thus aiding in establishing a stable cell line supporting FIPV replication. During the preparation of our manuscript, Schmied et al. reported the development of a stable cell line expressing serotype I FECV replicon with a GFP reporter gene and demonstrated its applicability as a screening platform for antiviral compounds [57]. The FECV replicon cDNA was cloned and inserted into a vaccinia virus vector using four steps of homologous recombination approach. The advantage of the vaccinia virus-based vector replicon is that it eliminates the need for multiple DNA/RNA preparation steps as a single plasmid system and facilitates the establishment of stable cell lines expressing the replicon. However, it involves complex handling of viral vectors, which requires expertise in generating recombinant vaccinia viruses and can be technically demanding. In contrast, the in vitro ligation replicon system is a technically straightforward approach, and it provides flexibility for simultaneously introducing multiple mutations and facilitating complex genetic studies. Nonetheless, it is labor-intensive, involving multiple steps of in vitro transcription and ligation, which can be time-consuming and less ideal for long-term studies than the single plasmid system. Moreover, the development of a stable cell line expressing serotype I FECV replicon by Schmied et al. is significant because it shows that stable cell lines can be generated without the need for specific mutations in the Nsps. This allows for further research to establish stable replicon-expressing cell lines for serotype II FCoV in various cell lines.

Despite extensive studies, the pathogenic and immunological mechanisms of FIP remain poorly understood, posing consistent challenges in clinical veterinary diagnosis and control [58]. The inability to grow serotype I field FECVs/FIPVs in standard cell culture systems poses a significant challenge to studies investigating the molecular pathogenesis of FIP [2]. Using reverse genetics technology, recombinant FIP viruses have been generated by combining the backbone of serotype I FECV/FIPV with the S protein of serotype II FIPV [26, 28]. These recombinant viruses have been experimentally confirmed to be capable of replicating in permissive cells in vitro and inducing infection in vivo. In addition, reverse genetics has also advanced the study of accessory proteins in FCoV. Previous research has demonstrated that the deletion of ORF3abc does not affect the replication of FIPV in vitro [18], which is consistent with our findings in this study. Conversely, ORF3abc plays a crucial role in the tropism of type II FIPV towards macrophages/monocytes [18]. Furthermore, another study showed that recombinant FIPV viruses lacking ORF7a exhibit a low replication rate in macrophages and have demonstrated that the absence of ORF7a leads to a significant attenuation in vivo [59]. The reverse genetics systems can be used to modify the target genes or sites via substitution, insertion, deletion, etc., followed by the reconstruction of recombinant infectious viruses, and subsequently analyse the phenotypes and biological properties of these modified viruses in cells or animal models to elucidate the functions of the target genes or sites, encompassing viral replication, infectivity, life cycle, and pathogenesis. Thus, we envisage that the reverse genetics system generated in this study will serve as a valuable tool in advancing our understanding of viral replication and the pathogenesis of FCoV. In addition, we demonstrated the use of recombinant reporter viruses as valuable screening tools for antiviral drugs, which offer advantages such as fluorescence or luminescence readouts for viral replication, surpassing labor-intensive methods like plaque assays or TCID_50_ quantification. The antiviral assay results showed consistent drug dose‒response curves between the recombinant reporter viruses (rFIPV-msfGFP and rFIPV-Rluc) and the wild-type FIPV WSU79-1184 against antiviral compounds, demonstrating the feasibility of reporter viruses as effective antiviral screening methods. Additionally, the stability of the rFIPV-msfGFP virus enables its utilization in long-term studies and in vitro experiments without the risk of losing its fluorescent marker.

Last, complementary to virus reverse genetic systems, replicon has been developed for many RNA viruses as a valuable tool [24, 60, 61]. Replicons have been utilized to investigate the molecular mechanisms underlying viral RNA synthesis and identify viral or cellular factors involved in the process. In this context, the role of specific Nsp, such as Nsp1, Nsp6, Nsp14, Nsp15, or Nsp16, in the replication of SARS-CoV and SARS-CoV-2 was examined without the need for infectious virus [62–64]. The replicon system is also helpful in elucidating the antiviral mechanisms of different antiviral compounds, thereby facilitating our discovery of drugs explicitly targeting viral genome replication.

In summary, we established a reverse genetics system for the wild-type FIPV WSU79-1146 strain, including reporter viruses and replicons. We believe the reverse genetics system developed here will provide a powerful tool for studying FCoV pathogenesis and identifying and characterizing future anti-FIPV compounds.

## Supplementary Information

**Additional file 1**: **Primers used in the construction of the recombinant viruses and the replicons.**

**Additional file 2**: **Assembly of the Full-Length rFIPV-WT, rFIPV-msfGFP, and rFIPV-Rluc cDNA.** **A** Gel analysis of the seven purified cDNA fragments. Individual fragments (**A**–**G**) were digested from corresponding plasmid clones and gel purified. Seven purified cDNA fragments were analysed on a 0.8% native agarose gel. The 1-kb DNA ladders are indicated. **B** Gel analysis of rFIPV-WT cDNA ligation products. Approximately 500 ng of purified ligation product was analysed on a 0.6% native agarose gel. The triangle indicates the full-length cDNA product. **C** Gel analysis of rFIPV-WT RNA transcripts. Approximately 1 μg of in vitro transcribed (IVT) RNAs were analysed on a 0.6% native agarose gel. The triangle indicates the genome-length RNA transcript. The circles show the shorter RNA transcripts. DNA ladders are indicated. Since this is a native agarose gel, the DNA size is not directly correlated to the RNA size. **D** Gel analysis of the purified cDNA G-msfGFP or G-Rluc fragments. Individual fragments were digested from corresponding plasmid clones and gel purified. The purified cDNA fragments were analysed on a 0.8% native agarose gel. The 1-kb DNA ladders are indicated. **E** Gel analysis of rFIPV-msfGFP cDNA ligation products. Approximately 500 ng of purified ligation product was analysed on a 0.6% native agarose gel. The triangle indicates the full-length cDNA product. **F** Gel analysis of rFIPV-Rluc cDNA ligation products. Approximately 500 ng of purified ligation product was analysed on a 0.6% native agarose gel. The triangle indicates the full-length cDNA product. **G** Gel analysis of rFIPV-msfGFP and rFIPV-Rluc RNA transcripts. Approximately 1 μg of IVT RNAs were analysed on a 0.6% native agarose gel. DNA ladders are indicated. The triangle indicates the genome-length RNA transcript. The circles show the shorter RNA transcripts. DNA ladders are indicated. Since this is a native agarose gel, the DNA size is not directly correlated to the RNA size.

**Additional file 3**: **NGS of the recovered recombinant viruses.** The sequencing depth and the mutational profiles across the genomes of **A** rFIPV-WT, **B** rFIPV-msfGFP, and **C** rFIPV-Rluc.

**Additional file 4:** **The condition optimization for high-throughput antiviral drug screening using rFIPV-msfGFP and rFIPV-Rluc.** **A** Left: Infection rate of rFIPV-msfGFP in different conditions. CRFK cells seeded in 96-well plates were infected with rFIPV-msfGFP at an MOI of 0.01 or 0.001 for 48 or 72 h. The uninfected cells served as mock control. Twelve technical replicates were performed for each condition. A high-content imaging readout was performed to count the cells containing msfGFP and calculate the infection rate of each replicate. Right: Z′ factor calculated from the infection rate of uninfected cells and infected cells under different conditions. **B** Left: The luciferase activity of rFIPV-Rluc under different conditions. CRFK cells seeded in 96-well plates were infected with rFIPV-Rluc at an MOI of 0.01 or 0.001 for 48 or 72 h. For each condition, cell lysate samples were subjected to luminescent analysis. The uninfected cells served as mock control. Twelve technical replicates were performed for each condition. Right: Z′ factor calculated from the luciferase activity of uninfected cells and infected cells at different conditions.

**Additional file 5**: **Correlation between inhibition rate and virus titre in the antiviral assay using rFIPV-msfGFP and rFIPV-Rluc.** Antiviral assay of GS-441524 or GC-376 using rFIPV-msfGFP **A** and Antiviral assay of GS-441524 or GC-376 using rFIPV-Rluc **B**. Relative inhibition was calculated based on the reporter gene expression in the cells treated with antiviral drugs, compared to cells treated with DMSO. The virus titre was measured by TCID_50_ assay. Error bars indicate means and standard deviations from three independent experiments.

**Additional file 6**: **Assembly of the repFIPV-msfGFP and repFIPV-Rluc cDNA.** **A** Gel analysis of the purified cDNA R-msfGFP and R-Rluc fragments. Individual fragments were digested from corresponding plasmid clones and gel purified. The purified cDNA fragments were analysed on a 0.8% native agarose gel. The 1-kb DNA ladders are indicated. **B** Gel analysis of repFIPV-msfGFP and repFIPV-Rluc ligation products. Approximately 500 ng of purified ligation product was analysed on a 0.6% native agarose gel. The triangle indicates the full-length replicon product. **C** Gel analysis of repFIPV-msfGFP and repFIPV-Rluc RNA transcripts. Approximately 1 μg of in vitro transcribed (IVT) RNAs were analysed on a 0.6% native agarose gel. The triangle indicates the genome-length RNA transcript. The circles show the shorter RNA transcripts. DNA ladders are indicated. Since this is a native agarose gel, the DNA size is not directly correlated to the RNA size.

## Data Availability

All the data generated in this study are included in the published article. The sequences described in this study have been deposited in GenBank under accession numbers OQ311323-OQ311325.

## Abbreviations

BAC: Bacterial artificial chromosome
CRFK: Crandell-Rees feline kidney
CPE: Cytopathic effect
DAPI: 4′,6-diamidino-2-phenylindole
DMEM: Dulbecco’s modified eagle medium
DMSO: Dimethylsulfoxide
E: Envelope
FBS: Fetal bovine serum
FCoV: Feline coronavirus
FECV: Feline enteric coronavirus
FIP: Feline infectious peritonitis
FMDV: Foot-and-mouth disease virus
hpi: Hours post-infection
HTS: High-throughput screening
IVT: In vitro transcription
M: Membrane
MERS: Middle East respiratory syndrome
MOI: Multiplicity of infections
msfGFP: Monomeric superfold green fluorescent protein
MTT: 3-[4,5-dimethylthiazol-2-yl]-2,5 diphenyl tetrazolium bromide
N: Nucleocapsid
NeoR: Neomycin resistance gene
Nsp: Non-structural proteins
ORF: Open reading frame
PBS: Phosphate-buffered saline
RdRp: RNA-dependent RNA-dependent RNA polymerase
RLU: Relative light units
Rluc: *Renilla* luciferase
RT: Reverse transcription
S: Spike glycoprotein
SARS: Severe acute respiratory syndrome
TAR: Transformation-associated recombination
TRS: Transcription regulatory sequence
YAC: Yeast artificial chromosome
EC_50_: 50% Maximal effective concentration
