# Correction: Development and characterization of reverse genetics systems of feline infectious peritonitis virus for antiviral research

**DOI:** 10.1186/s13567-025-01498-9

**Published:** 2025-03-24

**Authors:** Guoqian Gu, To Sing Fung, Wong Tsz Hung, Nikolaus Osterrieder, Yun Young Go

**Affiliations:** 1 Department of Infectious Diseases and Public Health, Jockey Club College of Veterinary Medicine and Life Sciences, City University of Hong Kong, Hong Kong, SAR China; 2 Department of Biomedical Sciences, Jockey Club College of Veterinary Medicine and Life Sciences, City University of Hong Kong, Hong Kong, SAR China; 3 Institut Für Virologie, Freie Universität Berlin, 14163 Berlin, Germany; 4 College of Veterinary Medicine, Konkuk University, Seoul, Republic of Korea

**Correction: Veterinary Research (2024) 55:124** 10.1186/s13567-024-01373-z

Following publication of the original article [1], the authors identified the following errors:

1. The fourth sentence states in the abstract: “In this study, a full-length infectious cDNA clone of the wild-type FIPV WSU79-1149 strain was constructed to generate recombinant FIPV (rFIPV-WT)…….”. The strain name should be corrected to **FIPV WSU79-1146**.
2. In the introduction, the first sentence of the third paragraph reads: “In this study, we constructed a full-length infectious cDNA of FIPV WSU79-1149 (serotype II)……” This should also be amend to **WSU79-1146**.
3. In the “Materials and Methods” section, under “Construction of infectious cDNA clones of rFIPV-WT, rFIPV-msfGFP, and rFIPV-Rluc,” the fifth sentence mentions: “The FIPV WSU79-1149 genome contained seven endogenous *BsaI* restriction………”. This should be updated to **WSU79-1146.**

The original article has been corrected.
