# Supplementary material for: Development and characterization of reverse genetics systems of feline infectious peritonitis virus for antiviral research
Source: Vet Res. 2024 Sep 27;55:124. doi: 10.1186/s13567-024-01373-z (PMC11438400; doi:10.1186/s13567-024-01373-z)
Supplement: Supplementary file 1 — Additional file 1: Primers used in the construction of the recombinant viruses and the replicons. [file 13567_2024_1373_MOESM1_ESM.docx]

**Additional file 1 Primers used in the construction of the recombinant viruses (rFIPV-WT, rFIPV-msfGFP, rFIPV-Rluc) and the replicons (repFIPV-msfGFP, repFIPV-Rluc)**

| **Primer** | **Sequence (5′ to 3′) *** | **Use** |
| --- | --- | --- |
| p14-A-fwd | CCCTG***GAGACC***TAATACGACTCACTATAGGA CTTTTAAAGTAAAGTGAGTGTAGCGTG | Amplify fragment A |
| p15-A-rev | GAA***GGTCTC***CGCACTTAGCACTAAGTGTGAC | Amplify fragment A |
| p16-B-fwd | GAA***GGTCTC***TGTGCGGCTGTGGTGTTAAAG | Amplify fragment B |
| p17-B-rev | CACATCAGGGATT***GGTCTC***ATATTACC | Amplify fragment B |
| p18-C-fwd | GGTAATAT***GAGACC***AATCCCTGATGTG | Amplify fragment C |
| p19-C-rev | GAA***GGTCTC***GCATCCTTAACCTCAACAATTGACC | Amplify fragment C |
| p20-D-fwd | GAA***GGTCTC***GGATGCTAATGGCGCTCAAG | Amplify fragment D |
| p21-D-rev | CATAAAAGT***GGTCTC***CTAAGACAGTCTC | Amplify fragment D |
| p22-E-fwd | GAGACTGTCTTAG***GAGACC***ACTTTTATG | Amplify fragment E |
| p23-E-rev | GAA***GGTCTC***ACCAGGGTTCCATTCAGCAGA | Amplify fragment E |
| p24-F-fwd | GAA***GGTCTC***CCTGGCTATAGCATGCCTACA | Amplify fragment F |
| p25-F-rev | CGAATCTCTGAGACT***GAGACC***TAACAC | Amplify fragment F |
| p26-G-fwd | GTGTTA***GGTCTC***AGTCTCAGAGATTC | Amplify fragment G |
| p27-G-rev | GAA***GGTCTC***TTTTTTTTTTTTTTTTTTTTTTTTTTTTTTTTGTGTATCACTATCAAAAGGAAAATTTTCAAACAAT | Amplify fragment G |
| p28-C1096T-fwd | CTTGTTGTGGTCT**T**CATGGCAAAGTTAAAG | C1096T mutagenesis |
| p29-C1096T-rev | AACTTTGCCATG**A**AGACCACAACAAGCAG | C1096T mutagenesis |
| p30-C16296G-fwd | AGTCGATGAGGT**G**TCTATGTGTACTAATTATGATC | C16296G mutagenesis |
| p31-C16296G-rev | AATTAGTACACATAGA**C**ACCTCATCGACTACAACAATG | C16296G mutagenesis |
| p32-C25037G-fwd | CTAAGCTTGGTCT**G**TCCTTTATTGAAGAAGTCA | C25037G mutagenesis |
| p33-C25037G-rev | CTTCTTCAATAAAGGA**C**AGACCAAGCTTAGCATACG | C25037G mutagenesis |
| p34-C27175T-fwd | ATGTCCGAGAGA**T**CTTGTTCCCAAAGG | C27175T mutagenesis |
| p35-C27175T-rev | CTTTGGGAACAAG**A**TCTCTCGGACATAAATTC | C27175T mutagenesis |
| p36-T7-N-fwd | TACTGTAATACGACTCACTATAGGGATAATACCACC  ATGGCCACACAGGGACAAC | Produce N-encoding amplicon as the template for IVT of N RNA |
| p37-N-A35-rev | TTTTTTTTTTTTTTTTTTTTTTTTTTTTTTTTTTTTTAG  TTCGTAACCTCATCAATCATCTCAAC | Produce N-encoding amplicon as the template for IVT of N RNA |
| p53-ORF3a-rev | TGACGATATCCATACAAGACCTGTAATGACTAATA AGTTTAGTTC | Construct rFIPV-msfGFP |
| p54-ORF3c-fwd | GCTTGCAGTTGAACTTCTCAATGGTGAT | Construct rFIPV-msfGFP |
| p55-msfGFP-fwd | GTCATTACAGGTCTTGTATGGATATCGTCAGTAAA GGTGAAGAACTCTTCACTGGAGT | Construct rFIPV-msfGFP |
| p56-msfGFP-rev | CCATTGAGAAGTTCAACTGCAAGCTTACTTGTACA GCTCATCCATACCGAG | Construct rFIPV-msfGFP |
| p106-hRluc-fwd | ACAGGTCTTGTATGGATATCATGGCTTCCAAGGTGTACGAC | Construct rFIPV-Rluc |
| p107-hRluc-rev | GAAGTTCAACTGCAAGCTTACTGCTCGTTCTTCAGCACG | Construct rFIPV-Rluc |
| p108-G549A-fwd | TTCTTCGTCGAAACCATGCTCCCAAGC | Construct rFIPV-Rluc |
| p109-G549A-rev | TTGGGAGCATGGTTTCGACGAAGAAG | Construct rFIPV-Rluc |
| p3-FIPV-Siddel-rev | GGAAGGTTCATCTCCCCAGT | Sequence rFIPV-msfGFP and rFIPV-Rluc |
| p79 -24611-fwd | GCCTTGGTATGTGTGGCTAC | Sequence rFIPV-msfGFP and icFIPV-Rluc |
| p95-msfGFP-255-fwd | CAAGTCTGCAATGCCTGAAG | Sequence rFIPV-msfGFP |
| p110-hRluc-417-fwd | CATCGTCCATGCTGAGAGTG | Sequence rFIPV-Rluc |
| p102-nsp16-rev | ACGGATCCACTCATGGCACATTAACTAAATGATTACCAAAGTT | Construct repFIPV-msfGFP |
| p103-msfGFP-fwd | TTAATGTGCCATGAGTGGATCCGTGAGTAAAGGTG | Construct repFIPV-msfGFP |
| p104-msfGFP-rev | TAGATTCTCGAGTCACTTGTACAGCTCATCCATACCG | Construct repFIPV-msfGFP |
| p105-M-fwd | AGCTGTACAAGTGACTCGAGAATCTAAAGCTGGTGATTACTCAACAG | Construct repFIPV-msfGFP |
| p114-GSG-F2A-fwd | GGATCCGGAGTGAAACAGACTTTGAATTTTGACCTTCTCAAGTTGGCGGGAGACGTC | Construct repFIPV-Rluc |
| p115-F2A-Neo-fwd | AAGTTGGCGGGAGACGTCGAGTCCAACCCTGGGCCCATGATTGAACAAGATGGATTGCA | Construct repFIPV-Rluc |
| p116-Neo-rev | CCAGCTTTAGATTCTCGAGTCAGAAGAACTCGTCAAGAAGGCG | Construct repFIPV-Rluc |
| p117-hRluc-fwd | ATGTGCCATGAGTGGATCTATGGCTTCCAAGGTGTACGAC | Construct repFIPV-Rluc |
| p118-hRluc-rev | CAAAGTCTGTTTCACTCCGGATCCCTGCTCGTTCTTCAGCACG | Construct repFIPV-Rluc |

* *BsaI* sites were italicized. Bases for mutagenesis in the OE-PCR primers were underlined.
