# Supplementary figures and images for: Development and characterization of reverse genetics systems of feline infectious peritonitis virus for antiviral research
Source: Vet Res. 2024 Sep 27;55:124. doi: 10.1186/s13567-024-01373-z (PMC11438400; doi:10.1186/s13567-024-01373-z)

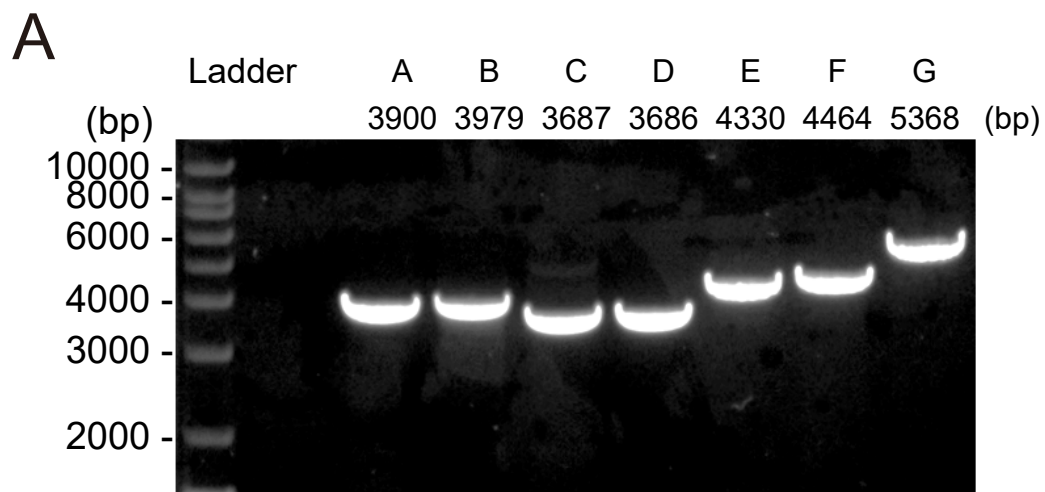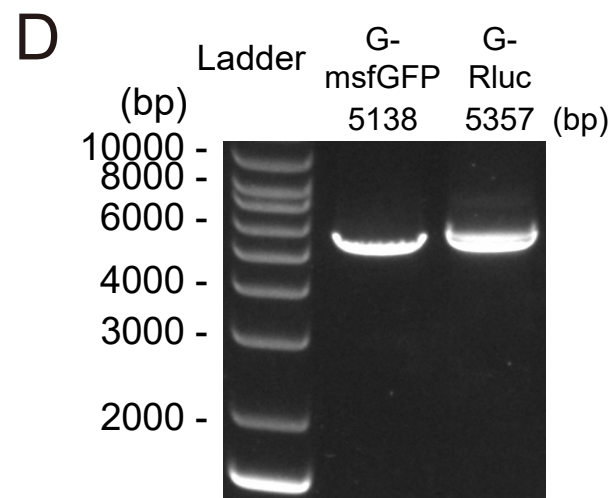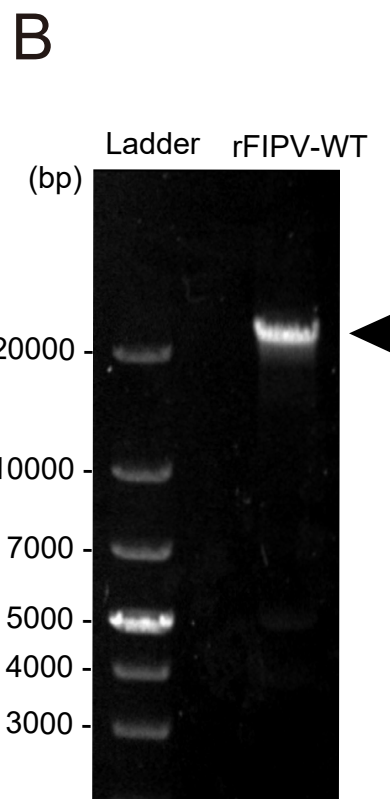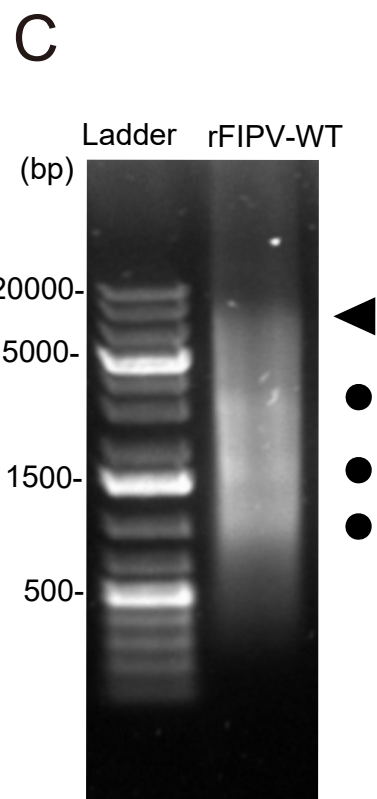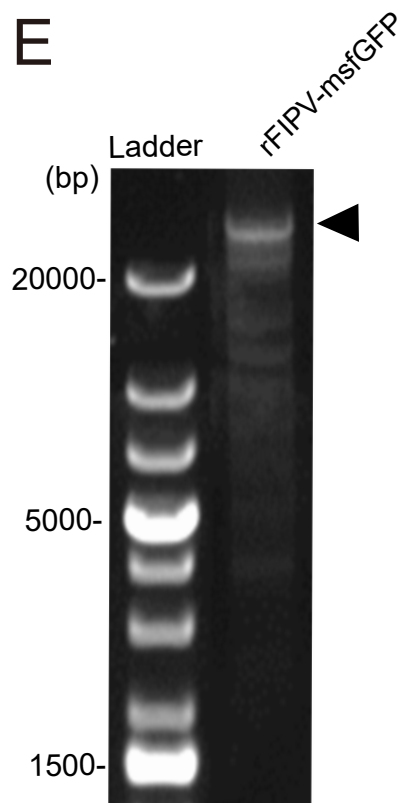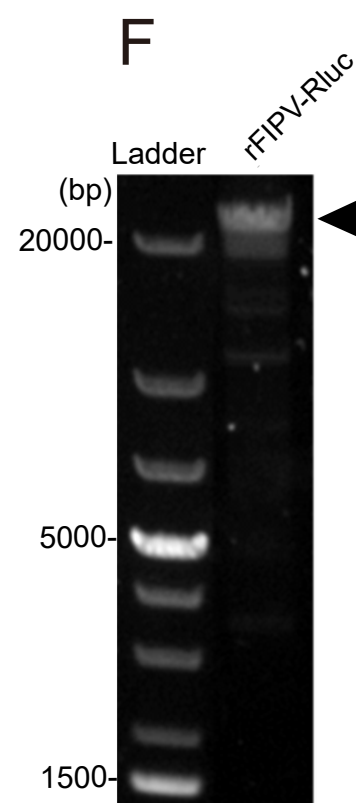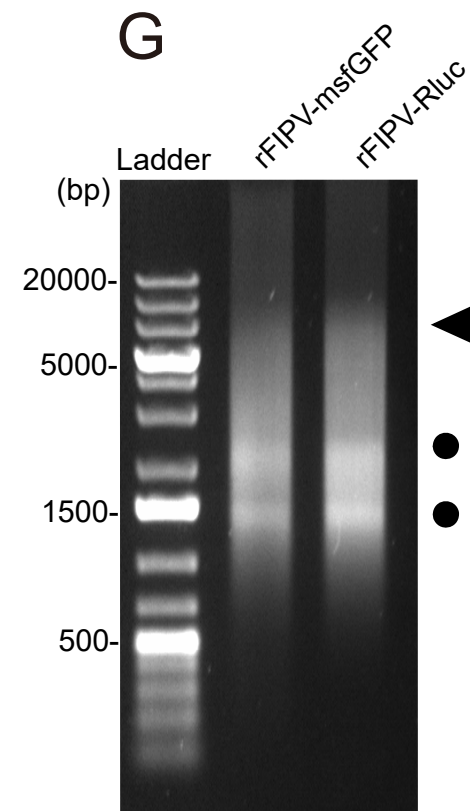

Supplement: Supplementary file 2 — Additional file 2: Assembly of the Full-Length rFIPV-WT, rFIPV-msfGFP, and rFIPV-Rluc cDNA. A Gel analysis of the seven purified cDNA fragments. Individual fragments (A–G) were digested from corresponding plasmid clones and gel purified. Seven purified cDNA fragments were analysed on a 0.8% native agarose gel. The 1-kb DNA ladders are indicated. B Gel analysis of rFIPV-WT cDNA ligation products. Approximately 500 ng of purified ligation product was analysed on a 0.6% native agarose gel. The triangle indicates the full-length cDNA product. C Gel analysis of rFIPV-WT RNA transcripts. Approximately 1 μg of in vitro transcribed (IVT) RNAs were analysed on a 0.6% native agarose gel. The triangle indicates the genome-length RNA transcript. The circles show the shorter RNA transcripts. DNA ladders are indicated. Since this is a native agarose gel, the DNA size is not directly correlated to the RNA size. D Gel analysis of the purified cDNA G-msfGFP or G-Rluc fragments. Individual fragments were digested from corresponding plasmid clones and gel purified. The purified cDNA fragments were analysed on a 0.8% native agarose gel. The 1-kb DNA ladders are indicated. E Gel analysis of rFIPV-msfGFP cDNA ligation products. Approximately 500 ng of purified ligation product was analysed on a 0.6% native agarose gel. The triangle indicates the full-length cDNA product. F Gel analysis of rFIPV-Rluc cDNA ligation products. Approximately 500 ng of purified ligation product was analysed on a 0.6% native agarose gel. The triangle indicates the full-length cDNA product. G Gel analysis of rFIPV-msfGFP and rFIPV-Rluc RNA transcripts. Approximately 1 μg of IVT RNAs were analysed on a 0.6% native agarose gel. DNA ladders are indicated. The triangle indicates the genome-length RNA transcript. The circles show the shorter RNA transcripts. DNA ladders are indicated. Since this is a native agarose gel, the DNA size is not directly correlated to the RNA size. [file 13567_2024_1373_MOESM2_ESM.pdf]

A

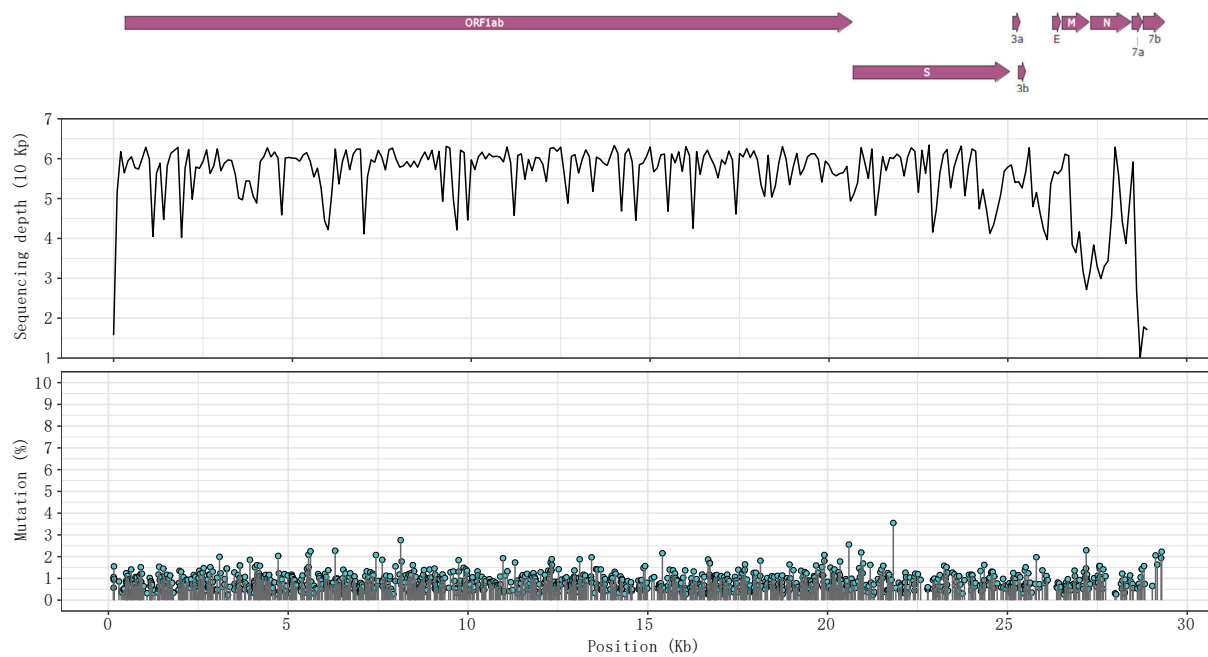

B

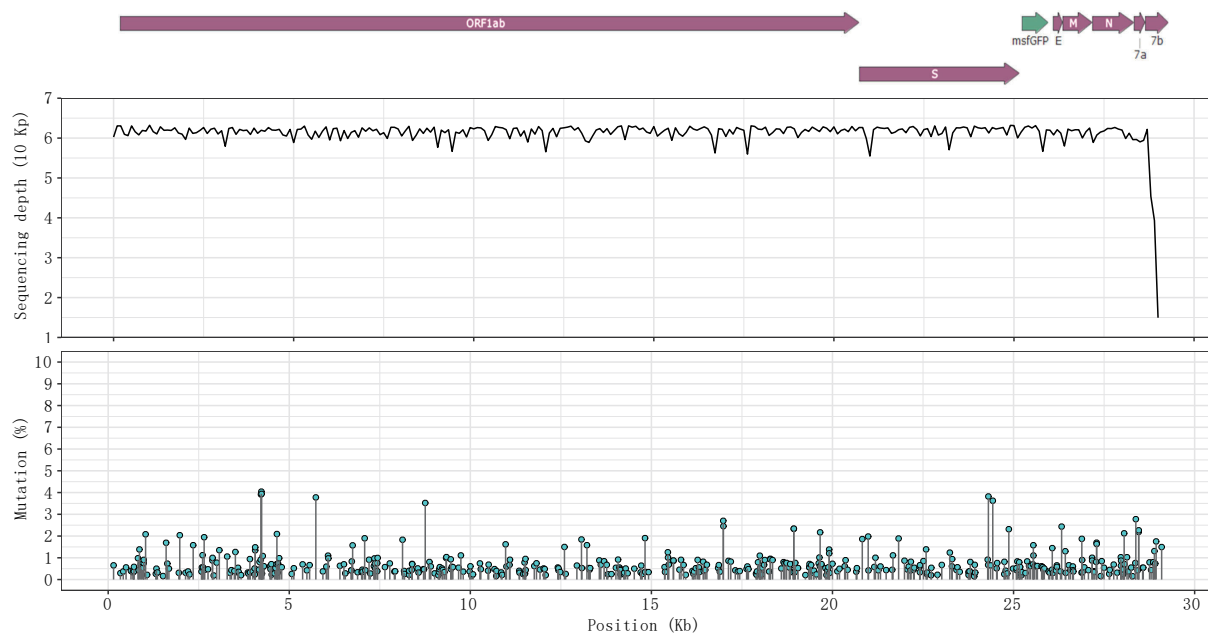

C

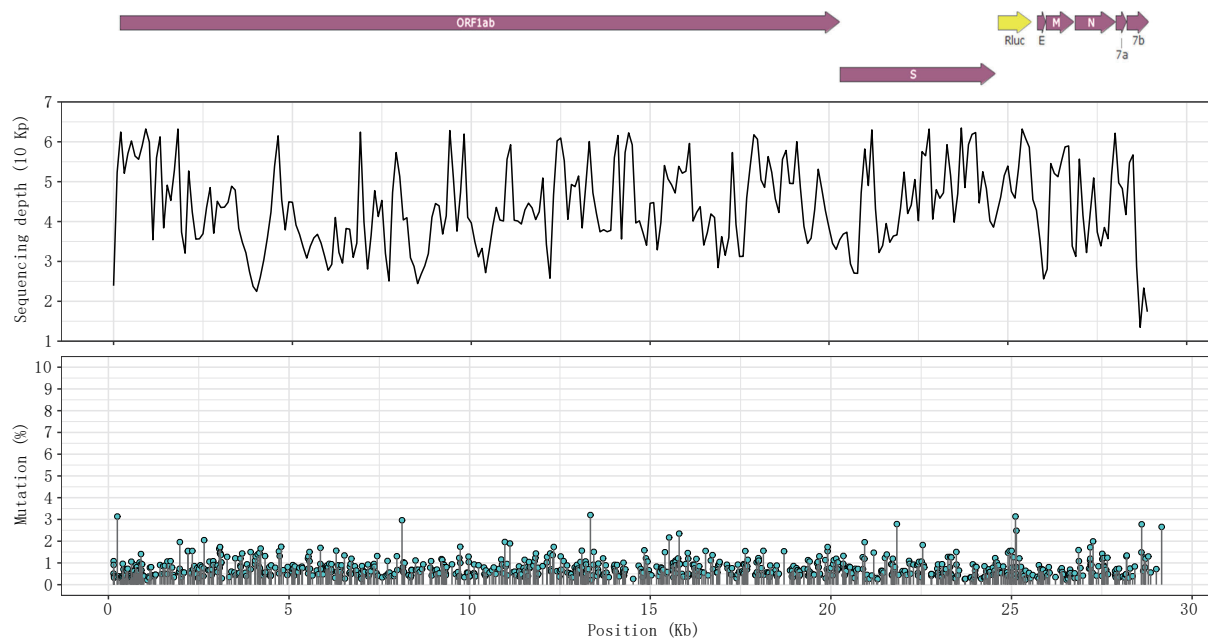

Supplement: Supplementary file 3 — Additional file 3: NGS of the recovered recombinant viruses. The sequencing depth and the mutational profiles across the genomes of A rFIPV-WT, B rFIPV-msfGFP, and C rFIPV-Rluc. [file 13567_2024_1373_MOESM3_ESM.pdf]

A

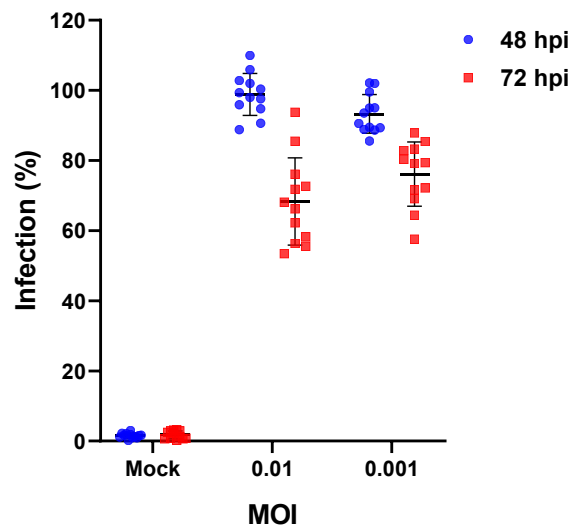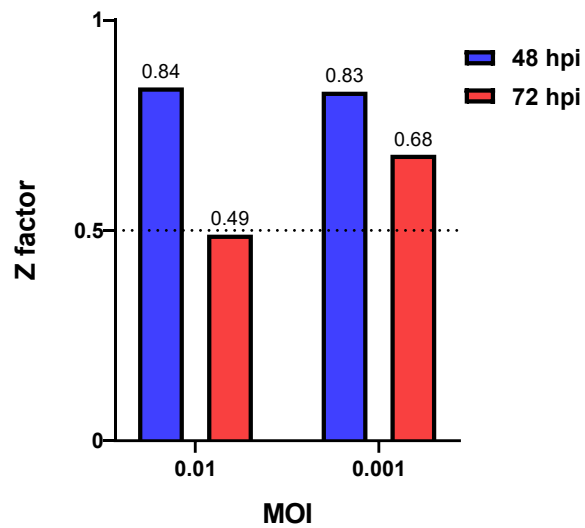

B

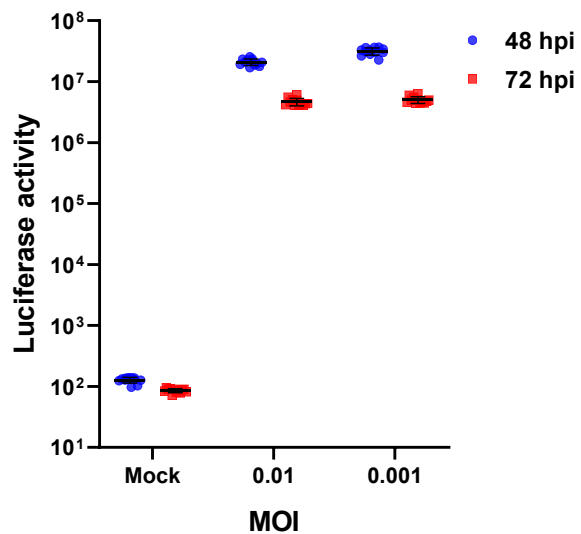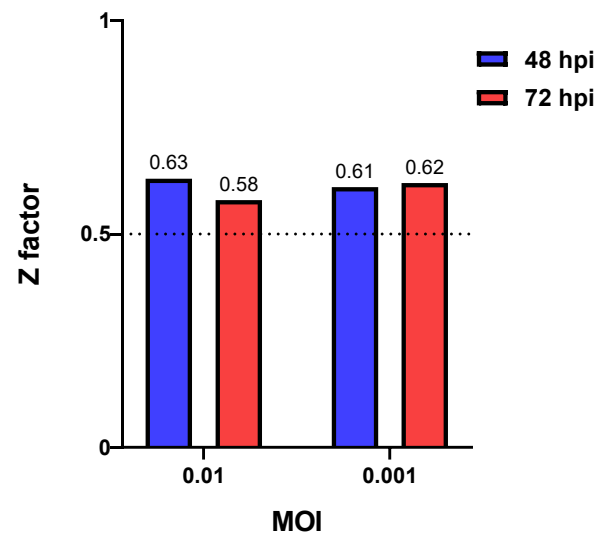

Supplement: Supplementary file 4 — Additional file 4: The condition optimization for high-throughput antiviral drug screening using rFIPV-msfGFP and rFIPV-Rluc. A Left: Infection rate of rFIPV-msfGFP in different conditions. CRFK cells seeded in 96-well plates were infected with rFIPV-msfGFP at an MOI of 0.01 or 0.001 for 48 or 72 h. The uninfected cells served as mock control. Twelve technical replicates were performed for each condition. A high-content imaging readout was performed to count the cells containing msfGFP and calculate the infection rate of each replicate. Right: Z′ factor calculated from the infection rate of uninfected cells and infected cells under different conditions. B Left: The luciferase activity of rFIPV-Rluc under different conditions. CRFK cells seeded in 96-well plates were infected with rFIPV-Rluc at an MOI of 0.01 or 0.001 for 48 or 72 h. For each condition, cell lysate samples were subjected to luminescent analysis. The uninfected cells served as mock control. Twelve technical replicates were performed for each condition. Right: Z′ factor calculated from the luciferase activity of uninfected cells and infected cells at different conditions. [file 13567_2024_1373_MOESM4_ESM.pdf]

A

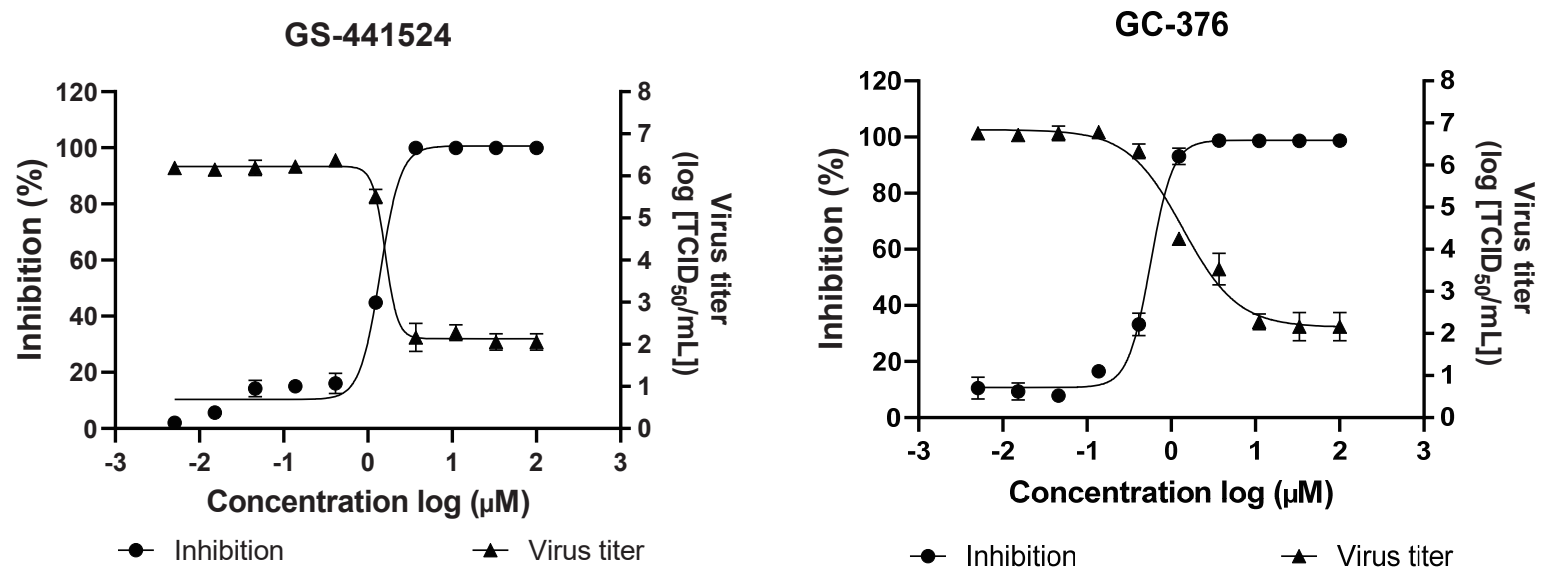

B

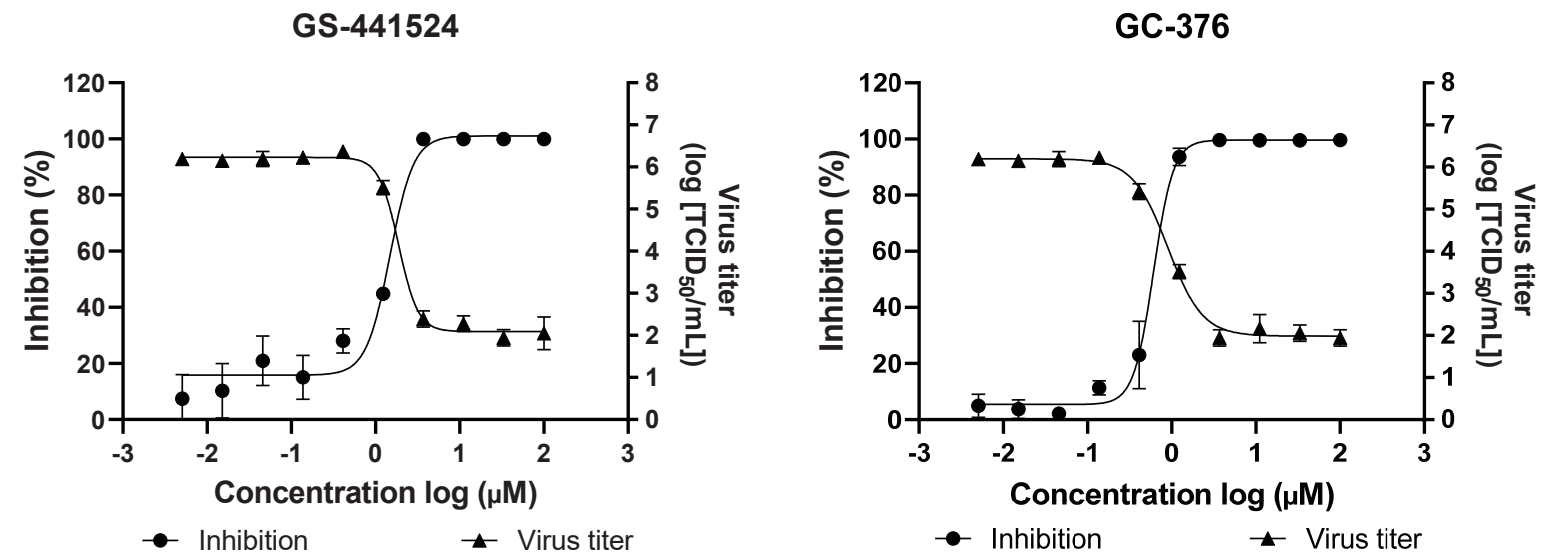

Supplement: Supplementary file 5 — Additional file 5: Correlation between inhibition rate and virus titre in the antiviral assay using rFIPV-msfGFP and rFIPV-Rluc. Antiviral assay of GS-441524 or GC-376 using rFIPV-msfGFP A and Antiviral assay of GS-441524 or GC-376 using rFIPV-Rluc B. Relative inhibition was calculated based on the reporter gene expression in the cells treated with antiviral drugs, compared to cells treated with DMSO. The virus titre was measured by TCID50 assay. Error bars indicate means and standard deviations from three independent experiments. [file 13567_2024_1373_MOESM5_ESM.pdf]

**A**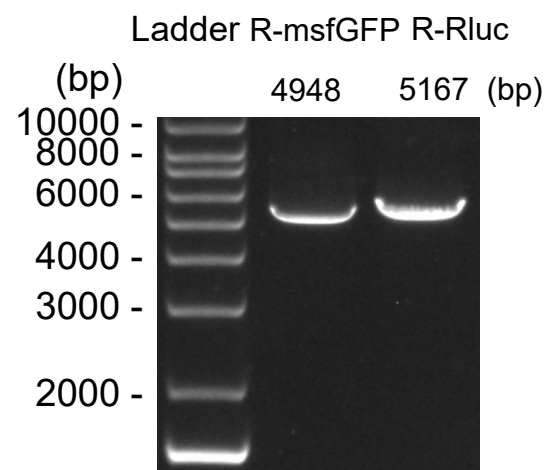**B**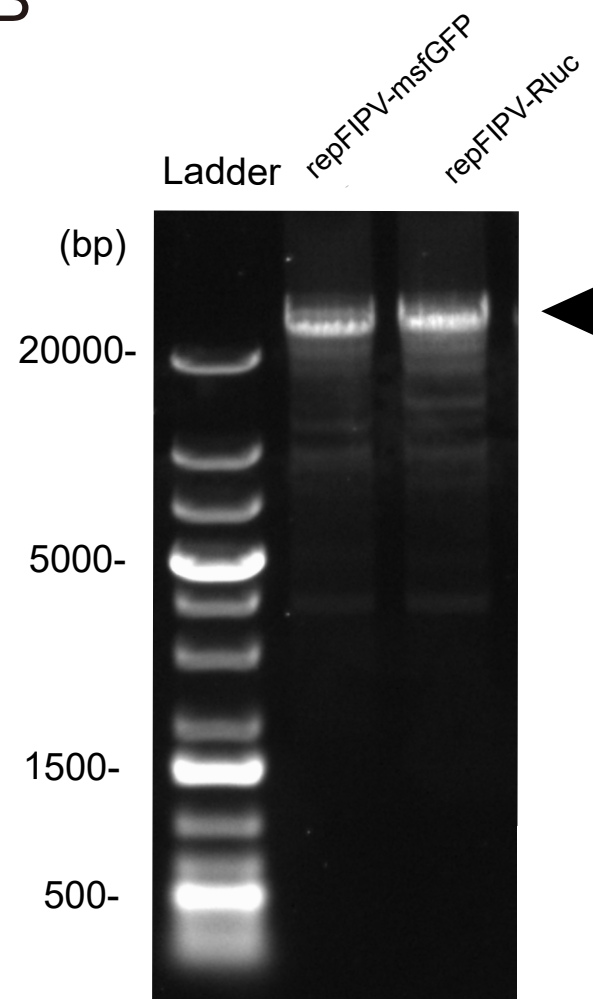**C**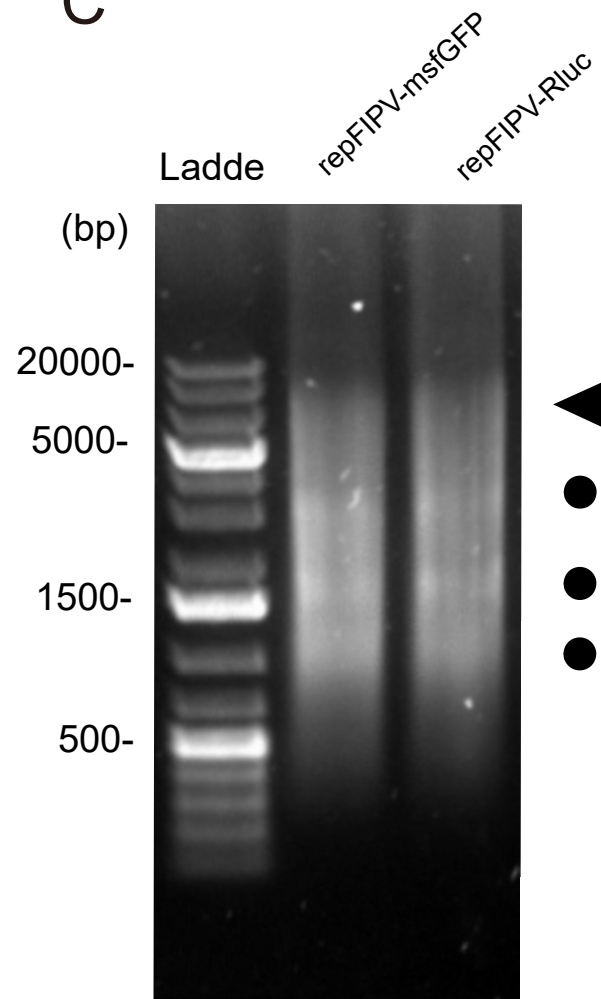

Supplement: Supplementary file 6 — Additional file 6: Assembly of the repFIPV-msfGFP and repFIPV-Rluc cDNA. A Gel analysis of the purified cDNA R-msfGFP and R-Rluc fragments. Individual fragments were digested from corresponding plasmid clones and gel purified. The purified cDNA fragments were analysed on a 0.8% native agarose gel. The 1-kb DNA ladders are indicated. B Gel analysis of repFIPV-msfGFP and repFIPV-Rluc ligation products. Approximately 500 ng of purified ligation product was analysed on a 0.6% native agarose gel. The triangle indicates the full-length replicon product. C Gel analysis of repFIPV-msfGFP and repFIPV-Rluc RNA transcripts. Approximately 1 μg of in vitro transcribed (IVT) RNAs were analysed on a 0.6% native agarose gel. The triangle indicates the genome-length RNA transcript. The circles show the shorter RNA transcripts. DNA ladders are indicated. Since this is a native agarose gel, the DNA size is not directly correlated to the RNA size. [file 13567_2024_1373_MOESM6_ESM.pdf]
